# Investigating the Relationships Between Basic Emotions and the Big Five Personality Traits and Their Sub‐Traits

**DOI:** 10.1111/jopy.13027

**Published:** 2025-05-15

**Authors:** Ryan Donovan, Aoife Johnson, Aine de Roiste, Ruairi O'Reilly

**Affiliations:** ^1^ Department of Psychology University of Galway Galway Ireland; ^2^ Department of Applied Social Studies Munster Technological University Cork Ireland; ^3^ Department of Computer Science Munster Technological University Cork Ireland

**Keywords:** basic emotions, big five aspects scale, emotion, five factor model, personality

## Abstract

**Introduction:**

Most research investigating relationships between the Big Five and emotional states has focused on how emotional attributes relate to Extraversion and Neuroticism. However, the potential for discrete emotional states to enable a richer understanding of the emotive nature of all Big Five traits and their subtraits has been neglected.

**Methods:**

Participants (*N* = 203) completed the Big Five Aspects Scale, watched six emotionally stimulating video clips, and self‐reported their experience of basic emotions before (Baseline) and after (Reaction) each video. Spearman correlations identified state–trait relationships, followed by regression analyses to assess the unique contribution of each trait to emotional experiences.

**Results:**

Conscientiousness negatively correlated with Baseline Sadness, while Agreeableness positively correlated with Reaction Disgust, Fear, and Sadness. Extraversion predicted higher Joy, and Neuroticism was linked to greater Fear and Sadness.

**Conclusion:**

Findings reinforce Extraversion and Neuroticism's links to positive and negative emotionality, respectively, while also showing that Agreeableness predicts heightened sensitivity to negative affect. Conscientiousness, particularly Orderliness, appears protective against Baseline Sadness, and Openness to Experience, especially Intellect, is linked to lower sensitivity to Surprise. Potential mechanisms underlying these relationships are discussed.

“Personality is to emotion as climate is to weather. That is, what one expects is personality, what one observes at any particular moment is emotion.” (Revelle and Scherer 2009, 304).

Personality and emotions are two of the most important concepts in psychological science. Personality is one's unique way of perceiving, feeling, and behaving in the world. Emotions are states of affect that influence how we perceive, behave, and feel in the world. Personality has been shown to significantly predict several important life outcomes, including one's health and well‐being, level of career and academic success, and quality of interpersonal relationships (Hudek‐Knežević and Kardum 2009; Malouff et al. 2010; Poropat 2009; Soto 2019). Healthy emotional experiences, expression, and awareness are associated with greater satisfaction in interpersonal relationships, a higher likelihood of attaining career success, and a lower likelihood of experiencing several clinical disorders (Graham et al. 2008; Hu et al. 2014; Urquijo et al. 2019). The respective foundations of these concepts are personality traits and basic emotional states.

Personality traits are stable patterns of affect, behavior, cognition, and motivation (Wilt and Revelle 2015). Personality traits differ across individuals but are stable within individuals throughout their lifespan. The leading model of personality traits in psychology is the Five‐Factor Model (FFM; John et al. 2008). The FFM identifies five broad factors of personality: Openness to Experience, Conscientiousness, Extraversion, Agreeableness, and Neuroticism. These five factors are known as the “Big Five” personality traits, as each trait is composed of granular sub‐traits, which are themselves composed of even more granular facets. The Big Five accurately predict life outcomes across several domains (health and well‐being, career and occupations, relationships, and education; see Soto 2019 for a review).

Basic emotions are a set of emotions that are (i) discrete, have unique and detectable psychological, behavioral, and neurophysiological signals, (ii) exist across cultures, and (iii) interact to form more complex and culturally mediated emotions (such as shame or guilt; Ekman 2004). While there is debate on the number of basic emotions, most basic emotion theory researchers agree on the following six emotions: Anger, Disgust, Fear, Joy, Sadness, and Surprise (Ekman and Cordaro 2011). The properties of these basic emotions (discrete, reliable signals, universality) enable a scientific analysis of both general emotional experiences and how they relate to other psychological constructs, such as personality.

Personality and emotions have long been considered related. The ancient Greek physician Galen formulated the earliest known model of personality, which identified four separate personality types: sanguine, choleric, melancholic, and phlegmatic (Revelle and Scherer 2009). Each type was associated with certain emotional states (e.g., melancholic personality types experienced more negative emotion, whereas sanguine personality types experienced more positive emotion). Similarly, emotional experiences are regularly described as one of the key components in definitions of personality traits (i.e., enduring patterns of affect). In affective neuroscience research, personality has been linked with key emotional networks identified in the central nervous system (Davis and Panksepp 2018). Finally, the relationship between personality and emotion was investigated so frequently at one point that personality‐emotion research was declared an “identifiable sub‐discipline” of psychological science (Larsen and Ketelaar 1991, 132).

However, there is a limited number of studies that have empirically investigated relationships between FFM personality traits and basic emotional states. Most personality‐emotion research studies have investigated relationships between (i) FFM personality traits and dimensions of emotional experiences (such as arousal and valence) rather than discrete emotional states (Anglim et al. 2020), (ii) emotions and personality at the Big Five level of the FFM trait hierarchy rather than their sub‐traits or facets, and (iii) emotional experiences with Extraversion and Neuroticism as opposed to Openness to Experience, Conscientiousness, and Agreeableness (Fayard et al. 2012; Larsen and Ketelaar 1991). The limited scope of such investigations means that it is unclear how the two foundational constructs of personality and emotion are connected.

It is important to investigate relationships between FFM personality traits and basic emotional states as such states provide several conceptual advantages over the dimensional approach. Firstly, it enables the distinction between different emotional states with similar arousal and valence levels (Harmon‐Jones et al. 2017). For instance, it can be challenging to differentiate between Anger and Disgust using the dimensional approach, as both are characterized by high arousal and negative valence. However, methodologies developed within the Basic Emotion Theory framework allow for more precise discrimination between these states (Harmon‐Jones et al. 2017). Secondly, the basic emotion approach better predicts resulting behaviors when states share similar dimensional attributes. While Anger and Disgust may appear similar in terms of arousal and valence, they elicit different behavioral responses. Anger typically motivates approach behavior towards its source (Carver and Harmon‐Jones 2009), whereas Disgust prompts avoidance behavior away from its source (Rozin et al. 2008). These nuanced distinctions underscore the utility of the basic emotion approach in providing novel insights into the mappings between state–trait relationships and the Big Five personality traits.

Secondly, a deeper understanding of the affective aspects of several traits can be enabled. Personality‐emotion research has predominantly focused on Extraversion and Neuroticism over other FFM traits. One rationale for this narrow focus is the claim that Extraversion and Neuroticism are directly (i.e., “temperamentally”) linked to emotional experiences, whereas Openness to Experience, Conscientiousness, and Agreeableness are indirectly (i.e., “instrumentally”) linked to emotional experiences (Larsen and Ketelaar 1991). This claim was initially supported by results indicating that state–trait relationships identified for Extraversion and Neuroticism were robust across environments, whereas relationships identified for other FFM traits were environmentally dependent (Larsen and Ketelaar 1991). This claim, however, is contradicted by several studies that have identified robust state–trait relationships for Openness to Experience, Conscientiousness, and Agreeableness (DeNeve and Cooper 1998; Fayard et al. 2012). Some of these relationships demonstrate a similar magnitude of effect sizes reported for the “temperamental” relationships for Extraversion and Neuroticism. As such, there are clear empirical grounds for exploring Openness to Experience, Conscientiousness, and Agreeableness relationships with emotional states and re‐evaluating the nature of such relationships.

Thirdly, the sub‐trait level of the FFM hierarchy can provide novel insight into state–trait relationships. The sub‐traits are traits that exist below the Big Five and above the facets and have been put forward as a potential solution to the bandwidth‐fidelity dilemma in personality research (Cronbach and Gleser 1957; Soto and John 2017). The sub‐trait level identified by DeYoung et al. (2007) consists of 10 personality traits, and these traits have clarified relationships between personality and important life outcomes, including educational performance, political preference, and susceptibility to developing a clinical disorder (Allen et al. 2018; Hirsh et al. 2010; León et al. 2017; Sun et al. 2018).

However, the sub‐traits have not been implemented in understanding state–trait relationships. This is a missed opportunity, as the authors posit that sub‐traits will enable deeper analysis of well‐established state–trait relationships (e.g., Neuroticism and states like Fear and Sadness) by evaluating whether such relationships are consistent across sub‐traits. Similarly, the sub‐traits will enable a thorough investigation of whether there are significant emotional aspects to the traits Openness to Experience, Conscientiousness, and Agreeableness that have not been identified in the literature so far. Overall, incorporating sub‐traits into understanding state–trait relationships will preserve a broad understanding of the emotional dimensions of personality (high bandwidth) than is offered by the facets, whilst enabling greater specificity than when using the Big Five alone (high fidelity).

## 1 Mapping FFM Traits to Basic Emotional States

Despite the wealth of research on both Big Five personality traits and basic emotional states, the specific connections between these two phenomena are not well understood. This sub‐section synthesizes current key findings from the empirical literature to identify potential relationships and formulate testable hypotheses.

### 1.1 Openness to Experience

Openness to Experiences reflects the tendency to be intellectually curious, open‐minded, and interested in aesthetic or artistic experiences. The sub‐traits Intellect and Openness reflect the intellectual and aesthetic aspects of the trait respectively (DeYoung 2015b; DeYoung et al. 2007). Openness to Experience is conceptualized as a cognitive disposition (see McCrae and Costa Jr. 1997; Saucier 1992 for reviews) and scant research has investigated the traits' relationship with basic emotional experiences. Research that has investigated its relationship with emotional experiences suggests that it is associated with both Disgust and Joy.

People high in Openness to Experience may be less likely to experience Disgust. It has been claimed that people high in Openness to Experience are more likely to take risks and expose themselves to potentially contaminating stimuli, whether physical (e.g., substances, new food) or psychological (e.g., new ideas, different cultures), which thereby makes them less likely to experience Disgust (Druschel and Sherman 1999; Haidt et al. 1994). This claim is supported by the finding that Openness to Experience is lower within populations of countries with higher rates of infectious diseases (*r* = −0.59; Schaller and Murray 2008). However, while a negative relationship between Openness to Experience and Disgust was found in a sample of 132 undergraduate students in the United States (Haidt et al. 1994), this result was not replicated in an Italian sample (Giampietro et al. 2019). The inconsistency of findings suggests that further research is required to evaluate the robustness of this hypothesized relationship.

People high in Openness to Experience may be more likely to experience Joy. In a meta‐analysis of 462 studies (*N* = 334,567), Openness to Experience positively correlated with positive affect (|*ρ*| = 0.24 Anglim et al. 2020). This replicates the positive correlation (*r* = 0.14) reported in the meta‐analysis conducted by DeNeve and Cooper (1998). Such findings constitute indirect evidence for a positive relationship between Openness to Experience and Joy, as it positively affects multiple aspects that are not related to Joy, such as alertness or engagement. This study will investigate the hypothesis that Openness to Experience is positively and specifically related to Joy.

### 1.2 Conscientiousness

Conscientiousness reflects the tendency to be disciplined and organized. Its two sub‐traits are Industriousness and Orderliness. Industriousness reflects its disciplined aspect, whereas Orderliness reflects its organized aspect (DeYoung 2015a; DeYoung et al. 2007). Conscientiousness is conceptualized as a behavioral and cognitive disposition. In two content analyses of the Big Five traits, Conscientiousness was found to have a high level of behavioral content (40.24%–67.80%), a moderate level of cognitive content (23.99%–26.10%), and a low level of affective content (6.20%–14.28%; see Wilt and Revelle 2015; Zillig et al. 2002). Despite this categorization, several studies have reported evidence of Conscientiousness having relationships with Anger, Disgust, Fear, and Sadness.

People high in Conscientiousness may be less likely to experience Anger, Fear, and Sadness. Conscientiousness negatively correlated with negative affect (|*ρ*| = |−0.25| − |−0.33|) across two meta‐analyses (Anglim et al. 2020; Fayard et al. 2012). Specific state–trait relationships were also investigated in the meta‐analysis reported by Fayard et al. (2012) and Conscientiousness was found to negatively correlate with Anger (*N* = 15,485, |*ρ*| = −0.23), Fear (*N* = 32,608, |*ρ*| = −0.22), and Sadness (*N* = 15,587, |*ρ*| = −0.26). Consequently, it is hypothesized that Conscientiousness will have a negative relationship with Anger, Fear, and Sadness.

Conscientious people may be more likely to experience Disgust. It is claimed that higher levels of Disgust sensitivity can make one more likely to keep their environments structured and to maintain discipline in their lives (Xu et al. 2019). Several empirical studies have supported this claim, reporting a positive correlation between Conscientiousness and Disgust (*r* = |0.11| − |0.28|; see Druschel and Sherman 1999; Giampietro et al. 2019; Tybur et al. 2009, 2011; Tybur and de Vries 2013). However, these results are contradicted by the negative correlation (*N* = 15,600, |*ρ*| = −0.21) found in the meta‐analysis of unpublished research conducted by Fayard et al. (2012), suggesting the presence of a publication bias effect. To investigate whether this is the case, this study will test the hypothesis that Conscientiousness has a positive relationship with Disgust.

## 2 Extraversion

Extraversion reflects the tendency to be gregarious and take charge of social situations. The two sub‐traits of Extraversion are Assertiveness and Enthusiasm. Assertiveness reflects the tendency to lead social situations, whereas Enthusiasm reflects the tendency to be gregarious. The relationship between Extraversion and emotional experiences has been well established. Extraverts have been shown to experience higher levels of Joy, with several studies demonstrating that Extraverts experience greater levels of global positive affect, daily positive affect, and momentary positive affect (Lucas and Baird 2004; Lucas et al. 2008; Smillie et al. 2015). The positive relationship has been identified across different measures, environments, and cultures (DeNeve and Cooper 1998; Joshanloo 2019; Watson and Clark 1992). Consequently, it is hypothesized that Extraversion will have a positive relationship with Joy.

## 3 Agreeableness

Agreeableness reflects the tendency to be prosocial and cooperative. The two sub‐traits of Agreeableness are Compassion and Politeness. Compassion reflects the tendency to be empathetic towards others, while Politeness reflects the tendency to be cooperative (DeYoung 2015b; DeYoung et al. 2007). Agreeable people may be less likely to experience Anger. Several findings support this claim. Agreeableness is negatively correlated with Anger across multiple lab‐based studies in Bresin et al. (2012), and people high in Agreeableness have been shown to be less likely to experience (i) daily Anger (*r* = −0.37), (ii) external expressions of Anger (*r* = −0.41), and (iii) internal expressions of Anger (*r* = −0.26; Özyeşil 2012). This relationship has been identified at the neurobiological level, as a meta‐analysis found Agreeableness negatively correlated with the ANGER/RAGE network (|*ρ*| = −0.41; Marengo et al. 2021).

These results have occasionally failed to replicate. One experimental study correlated Agreeableness with Anger measured before the study (Baseline) and after an experimental condition where Anger was induced (Reaction; Pfeiler et al. 2018). Agreeableness negatively correlated with Reaction Anger (*r* = −0.14) but had no significant correlation with Baseline Anger. Similarly, Agreeableness negatively correlated with external expressions of Anger (e.g., confrontation), but had no significant correlation with Anger experiences in the study reported by Sanz et al. (2010). This study aims to further investigate this relationship by testing the hypothesis that Agreeableness has a negative relationship with Anger.

## 4 Neuroticism

Neuroticism reflects the tendency to be emotionally reactive and self‐conscious. Its sub‐traits are Volatility and Withdrawal. Volatility reflects its emotionally reactive aspect, whereas Withdrawal reflects its self‐conscious aspect (DeYoung et al. 2007). Neuroticism is considered the Big Five trait with the most affect‐based components (Wilt and Revelle 2015; Zillig et al. 2002). Neuroticism has been shown to positively correlate with negative affect. The relationship between Neuroticism and negative affect is strong (|*ρ*| = 0.56; see Anglim et al. 2020). Consequently, Neuroticism has been labeled as the “Negative Affect “trait or as “Negative Emotionality” (Larsen and Ketelaar 1991). Concerning basic emotions, people high in Neuroticism are more likely to experience Fear and Sadness. Neuroticism is associated with higher sensitivity of activity in the neurological systems that govern these emotional experiences (Marengo et al. 2021) and regulate their expression (DeYoung and Allen 2018). Consequently, it is hypothesized that Neuroticism will have a negative relationship with Fear and Sadness.

## 5 The Present Study

This study investigates the research question: What are the relationships between FFM personality traits and basic emotional states? As far as the authors are aware, this is the first study to investigate relationships between all the Big Five traits, their subtraits, and the six basic emotional states. This study will also investigate whether such relationships are consistent pre (Baseline) and post (Reaction) emotion induction. This approach has been used in prior research when looking at specific state–trait relationships (e.g., Agreeableness and Anger in Pfeiler et al. (2018); however, this study reflects the first use of this approach for measuring the relationship between each basic emotion and Big Five trait. This aspect of the study enables the investigation of whether traits are related to heightened or reduced experience and sensitivity to emotional states, enhancing the scope of the work. The hypotheses being tested in this work are summarized in Table S1 within the Supporting Information.

## 2 Method

### 2.1 Participants

Participants were recruited using a convenience sampling approach between March 2021 and November 2021. An information sheet describing the study was shared both online (via LinkedIn, Facebook, Twitter, and Reddit) and with Psychology and Computer Science students at several third‐level institutions (Munster Technology University, University of Limerick, Technical University Darmstadt, Ulster University). Participants who completed the study received a personality report that described their FFM personality trait scores and were entered into a draw to win one of thirty €30 Amazon vouchers. Participants were recruited between May and December 2021. Statistical power analysis identified that a sample size of 152 participants was required to attain sufficient statistical power (1 − *β* = 80%) when trying to detect a moderate effect size (|*ρ*| = 0.20) for one‐tailed hypotheses (*α* error probability = 0.05). The study was approved by Munster Technological University's research ethics committee.

Overall, 203 participants were recruited. The mean age of participants was 34.26 (SD = 12.29, range = 18–70). Regarding gender, 107 participants identified as female (52.70%), 95 identified as male (46.80%), and 1 identified as non‐binary (0.50%). Participants came from diverse backgrounds, including the United Kingdom (*n* = 83), Ireland (*n* = 68), Germany (*n* = 25) Poland (*n* = 4), Portugal (*n* = 4), Russia (*n* = 2), South Africa (*n* = 2), India (*n* = 2), Slovenia (*n* = 1), United States (*n* = 3), Zambia (*n* = 1), Pakistan (*n* = 1), Macedonia (*n* = 1), Hungary (*n* = 1), Greece (*n* = 1), Canada (*n* = 1), Ukraine (*n* = 1), Philippines (*n* = 1), and Cambodia (*n* = 1). Most participants had achieved a bachelor's degree (*n* = 81), followed by a secondary school diploma (*n* = 48), master's degree (*n* = 40), and doctorate (*n* = 7). The education variable had some missing or erroneous data (*n* = 6); a manual check of the corresponding participants' data did not detect any other missing entries. All other participants completed either trade school or some level of secondary school.

### 2.2 Materials

All study materials are included as Supporting Information (see document S3_Study_Material).

#### 2.2.1 Measures

##### 2.2.1.1 Personality

The Big Five Aspects Scale (BFAS) was used to assess personality (DeYoung et al. 2007). The BFAS is a 100‐item questionnaire that measures the Big Five traits and their associated sub‐traits. The BFAS asks participants to rate how accurately items such as “I seldom feel blue” (Neuroticism reversed), “I often get lost in thought” (Openness to Experience), and “I carry out my plans” (Conscientiousness), describe their personality using a 1 (“Strongly Disagree”) to 5 (“Strongly Agree”) Likert scale. There are 20 items per each Big Five trait, which is further divided into 10 items for each of its two sub‐traits. Sub‐traits scores are calculated by computing the mean value for their 10 respective items. Big Five trait scores are calculated by computing the mean value for their 20 respective items. BFAS was selected as it is the only personality questionnaire that measures the Big Five sub‐traits identified by DeYoung et al. (2007). The Cronbach alpha scores are reported in Table 1.

**TABLE 1:** Descriptive statistics for the FFM personality traits.

Personality trait	M	SD	Median	Min	Max	Range	*α*
**Openness to experience**	3.73	0.53	3.70	1.90	5.00	3.10	0.83
Openness	3.68	0.66	3.70	1.80	5.00	3.20	0.80
Intellect	3.79	0.65	3.80	1.90	5.00	3.10	0.84
**Conscientiousness**	3.47	0.59	3.45	1.75	4.95	3.20	0.87
Industriousness	3.40	0.68	3.40	1.80	5.00	3.20	0.84
Orderliness	3.53	0.67	3.60	1.70	4.90	3.20	0.82
**Extraversion**	3.32	0.59	3.35	1.80	4.65	2.85	0.89
Assertiveness	3.17	0.73	3.20	1.20	4.80	3.60	0.88
Enthusiasm	3.48	0.65	3.50	1.40	4.80	3.40	0.83
**Agreeableness**	3.96	0.52	4.00	1.95	4.95	3.00	0.87
Compassion	4.03	0.68	4.10	1.40	5.00	3.60	0.89
Politeness	3.89	0.56	3.90	1.80	5.00	3.20	0.75
**Neuroticism**	2.88	0.65	2.90	1.25	4.50	3.25	0.89
Withdrawal	2.89	0.75	2.90	1.00	4.60	3.60	0.85
Volatility	2.84	0.77	2.80	1.10	4.90	3.80	0.87

##### 2.2.1.2 Emotion

A modified version of the Discrete Emotions Questionnaire (DEQ) was used to assess participants' basic emotional experiences (Harmon‐Jones et al. 2016). The DEQ is a 32‐item questionnaire that measures eight distinct emotional experiences (Anger, Anxiety, Disgust, Fear, Joy, Sadness, Surprise, and Relaxation). The scale was modified because the participants answered the scale seven different times during the study. The DEQ contains multiple items per construct (e.g., there are four DEQ items for Anger – “Anger”, “Rage”, “Mad”, and “Pissed Off”). To reduce participant load, the modified scale only included one item per basic emotional state, which were the item that directly named that state (i.e., Anger, Disgust, Fear, Joy, Sadness, and Surprise), and participants were asked to rate the extent they experienced each basic emotion using a 1 (“Not at all”) to 5 (“An extreme amount”) Likert scale.

#### 2.2.2 Stimuli & Tasks

##### 2.2.2.1 Emotional Stimuli

Video clips were used to induce emotional experiences. Video clips are the most effective inducers of basic emotional experiences within emotion induction research (Siedlecka and Denson 2019). In this research, 24 video clips were initially selected, with 4 video clips per basic emotion. These video clips were selected based on the research team's consensus and/or their proven efficacy in past research studies (see Gabert‐Quillen et al. 2015; Gilman et al. 2017). Two pilot studies were then conducted to identify the most effective video clip for inducing each basic emotion. The content of six video clips selected for this study is described in the Supporting Information (see Table S3 in document S3_Study_Material).

### 2.3 Design

An online correlational study design was employed to investigate the relationships between emotional states and personality traits. The Personality Emotion Mapping online application was used to host the research study and collect participants' data. The key variables to be measured were (a) the six self‐reported basic emotional states before participants began the study (Baseline condition), (b) the mean experience of the six basic emotional states across the six emotional stimuli (Reaction condition), and (c) the Big Five personality traits and their respective sub‐traits. The Big Five and their sub‐traits were correlated with each basic emotional state across Baseline and Reaction conditions.

A global approach was adopted to quantify the relationship between FFM personality traits and Reaction emotional states. The mean of each basic emotion across the six video clips per participant was computed before correlating this mean score with the participant's mean scores on the FFM personality traits.

A global approach was adopted for two reasons. Firstly, a global approach can avoid the issue of ceiling effects obscuring state–trait relationships. Each video clip selected is an effective inducer of a specific emotional state, making it more difficult to identify variances in state–trait relationships for that specific video. Secondly, a global approach is better equipped to capture diverse experiences to emotional stimuli. People can have several different emotional experiences while watching video clips (Larsen and Stastny 2011). If the analysis was limited to one emotional state per video, such diverse experiences would not be captured.

The study also involved the (i) video recording of participants' facial expressions while watching the video clips, (ii) audio recording of participants' reflections on the video clips guided by the speaking task, and (iii) transcription of said audio. Data from these three modalities (visual, audio, and text) was captured to enable multimodal analysis of state–trait relationships. Further information on the analysis of state–trait relationships across modalities can be found in Donovan (2023).

### 2.4 Procedure

Participants first read a participant information form that explained the purpose of the study, their rights as participants, and any potential benefits or risks of taking part in the study. Participants who consented to take part were then given a tutorial on how to navigate the PEM web application and how to test their audio and video before taking part.

Participants then completed a demographic questionnaire, the BFAS, the modified DEQ, and one random item from the Cognitive Reflection (Toplak et al. 2014) to act as a cognitive buffer. The order of the BFAS, DEQ, and CRT items was randomized per participant. The DEQ and CRT provided a baseline measure of the participants' cognitive and emotional state before they watched the video clips.

The video clip stage of the study consisted of six blocks. Each block contained a video clip, a DEQ scale, an audio prompt, and a CRT item. The order of blocks was randomized per participant. Participants were required to play the video clip in full before they could complete other tasks within the block. Before each video clip, participants were instructed to (i) watch the video clip in a quiet and well‐lit room, (ii) test the quality of their video and audio via the online application testing tool, (iii) sit directly in front of their web camera, and (iv) avoid obstructing their face while watching the video clip.

During each video clip, the participants' web camera was activated, and their video was recorded for the duration of the video clip. The recording immediately stopped once the video clip had ended. After each video clip, participants first answered the DEQ and then spoke about their emotional experiences for 1 min. The order in which participants answered the DEQ or spoke about their experiences was counterbalanced across the six videos. The CRT item was the final item for each block. Participants were unable to proceed to the next block until they had answered each item in the block. Participants were debriefed about the study and directed to internationally available emotional support services if they had been negatively affected by the study. The median time to complete the study was 70 min.

## 3 Results

All results, materials, and data are publicly available through the study's Open Science Framework directory: https://osf.io/rgac6/. The methodology and hypotheses were not pre‐registered before conducting the study or analysis. This study uses empirical benchmarks to evaluate effect sizes in psychological research in place of the traditional practice of Cohen's rules of thumb (Funder and Ozer 2019). These benchmarks identify the strength of an effect for explaining single events, with *r* = |0.05| − |0.09| as very small, *r* = |0.10| − |0.19| as small, *r* = |0.20| − |29| as medium, *r* = |0.30| − |0.39| as large, and *r* ≥ |0.40| as very large. Pairwise correlation between all personality and emotion variables can be found in the Supporting Information (Table S2).

### 3.1 Descriptive Statistics

#### 3.1.1 Personality and Emotion

Table 1 presents key descriptive statistics on personality scores. Table 2 presents the descriptive statistics that were computed for participants' responses to each basic emotion across the 7 DEQ questionnaires (1 baseline; 6 reactions). Table 3 presents the mean emotion response per video clip, showing that for three of the six basic emotions, at least one of the video clips elicited a strong emotional response (> 4).

**TABLE 2:** Descriptive statistics for the six basic emotions, before (baseline) and after (reaction) emotion induction.

Statistics	Anger	Anger	Disgust	Disgust	Fear	Fear
	Baseline	Reaction	Baseline	Reaction	Baseline	Baseline
*N*	203	203	203	203	203	203
Mean	1.51	1.96	1.32	2.52	1.51	2.12
SD	0.75	0.52	0.68	0.58	0.82	0.58
Median	1.00	2.00	1.00	2.50	1.00	2.00
Min	1.00	1.00	1.00	1.17	1.00	1.00
Max	4.00	3.33	5.00	3.83	5.00	3.67
Range	3.00	2.33	4.00	2.67	4.00	2.67

	Joy	Joy	Sadness	Sadness	Surprise	Surprise
	Baseline	Reaction	Baseline	Reaction	Baseline	Reaction
*N*	203	203	203	203	203	203
Mean	3.15	1.99	1.77	2.30	1.84	2.88
SD	0.96	0.56	0.96	0.55	1.02	0.83
Median	3.00	2.00	2.00	2.33	1.00	2.83
Min	1.00	1.00	1.00	1.00	1.00	1.00
Max	5.00	3.33	5.00	3.83	5.00	5.00
Range	4.00	2.33	4.00	2.83	4.00	4.00

*Note:* Rows and columns are transposed to facilitate a comparison between the Baseline and Reaction experience for each emotion.

**TABLE 3:** Mean emotion per video clip across sample.

Video^a^	Anger		Disgust		Fear		Joy		Sadness		Surprise	
	M	SD	M	SD	M	SD	M	SD	M	SD	M	SD
AN	1.56	0.98	2.07	1.18	**3.94**	1.11	1.22	0.62	1.64	0.88	3.35	1.22
HMS	1.17	0.56	1.37	0.78	1.13	0.51	**3.24**	1.28	1.12	0.44	2.61	1.35
LK	2.80	1.29	2.20	1.31	2.62	1.19	1.66	0.98	**4.08**	1.09	1.86	1.02
SL	3.72	1.30	**3.99**	1.18	2.38	1.28	1.05	0.27	3.81	1.18	2.58	1.32
TS	1.41	0.83	**4.38**	1.07	1.54	0.85	2.12	1.20	1.79	1.10	2.79	1.32
WD	1.09	0.33	1.11	0.44	1.17	0.56	2.83	1.36	1.43	0.84	**4.21**	1.03

*Note:* The scores that are in bold indicate the strongest emotional responses elicited by each video clip.

Abbreviations: AN, Annabelle; HMS, When Harry Met Sally; LK, The Lion King; SL, Schindler's List; TS, Trainspotting; WD, Test Your Awareness—WhoDunnit?

^a^ Acronyms are used in place of the full title to keep the table within the column margins.

### 3.2 Correlation Between Baseline and Reaction Emotional States

Spearman rank‐order correlations were computed to identify relationships between Baseline and Reaction‐based emotional experience (Supporting Information, Table S2). This test was selected because while all our variables were scale data and most were normally distributed, some violated normality (Shapiro‐Wilks test, *p* ≤ 0.05). There were small‐to‐large positive correlations between Baseline and Reaction emotional experiences for Anger (*p* ≤ 0.001), Fear (*p* = 0.05), and Sadness (*p* ≤ 0.001). Baseline Disgust had a non‐significant correlation with Reaction Disgust (*p* = 0.45) and Baseline Surprise had a non‐significant correlation with Baseline Surprise (*p* = 0.08).

### 3.3 Correlating FFM Personality Traits and Basic Emotions

Spearman's rank‐order correlation was computed to assess the between the Big Five traits and their 10 sub‐traits with both Baseline and Reaction basic emotions. To ensure consistency and handle non‐normal data, Spearman's correlation was applied throughout, offering a robust, non‐parametric measure valid for both normal and non‐normal continuous variables. Tables 4 and 5 show the correlation matrix between FFM personality traits and basic emotional experiences per Baseline and Reaction conditions. Given that this research was exploratory in its aims, no multiple comparison corrections (e.g., Bonferroni) were applied to account for the number of analyses. This decision was made to reduce the risk of Type II errors and ensure that potentially meaningful state–trait relationships were not overlooked. However, this approach increases the likelihood of Type I errors. Consequently, all significant findings warrant further investigation to assess their replicability.

**TABLE 4:** Correlation matrix between FFM personality traits and baseline basic emotions.

	Anger	Disgust	Fear	Joy	Sadness	Surprise
Openness to Experience	−0.05	−0.05	−0.01	−0.03	0.01	0.10
Intellect	−0.07	−0.08	−0.10	0.03	−0.13	0.09
Openness	−0.01	0.01	0.06	−0.03	**0.** **17** *	0.07
Conscientiousness	**−0.15** *	**−0.18** **	**−0.17** *	0.08	**−0.30** ***	0.00
Industriousness	**−0.22** ***	**−0.19** **	**−0.25** ***	0.08	**−0.32** ***	0.00
Orderliness	−0.04	−0.11	−0.02	0.05	**−0.21** **	0.00
Extraversion	0.04	0.02	0.12	**0.** **23** **	−0.08	0.08
Assertiveness	0.04	−0.03	**−0.16** *	0.13	−0.13	0.06
Enthusiasm	0.02	0.05	−0.03	**0.** **28** ***	−0.02	0.07
Agreeableness	−0.05	−0.05	0.13	0.06	0.05	0.00
Compassion	0.06	0.04	0.11	0.08	0.11	0.05
Politeness	**−0.17** *	**−0.15** *	0.11	0.01	−0.06	−0.06
Neuroticism	**0.** **27** ***	0.00	**0.** **30** ***	−0.12	**0.** **28** ***	−0.10
Volatility	**0.** **26** ***	0.07	**0.** **18** **	−0.11	**0.** **22** **	−0.08
Withdrawal	**0.** **26** ***	0.01	**0.** **40** ***	−0.11	**0.** **32** ***	−0.09

* *p* ≤ 0.05.

** *p* ≤ 0.01.

*** *p* ≤ 0.001.

**TABLE 5:** Correlation matrix between FFM personality traits and reaction basic emotions.

	Anger	Disgust	Fear	Joy	Sadness	Surprise
Openness to Experience	−0.09	−0.02	−0.01	−0.01	0.02	−0.07
Intellect	−0.05	−0.02	−0.07	0.01	−0.02	**−0.14** *
Openness	−0.06	0.02	0.07	−0.01	0.07	0.03
Conscientiousness	0.11	0.00	−0.06	**−0.22** **	−0.03	0.01
Industriousness	0.07	−0.03	−0.10	**−0.15** *	−0.05	−0.02
Orderliness	0.12	0.04	0.00	**−0.24** ***	−0.01	0.00
Extraversion	**0.** **17** *	**0**.**15** *	0.12	**0**.**18** **	0.13	0.08
Assertiveness	0.12	0.08	0.01	**0.** **15** *	0.05	0.03
Enthusiasm	**0.** **15** *	**0.** **15** *	**0.** **18** **	0.13	**0.** **15** *	0.09
Agreeableness	0.08	**0.** **17** *	**0.** **27** ***	−0.08	**0.** **19** **	0.10
Compassion	0.13	**0.** **20** **	**0.** **32** ***	−0.02	**0.** **24** ***	0.12
Politeness	−0.01	0.06	0.11	**−0.14** *	0.04	0.09
Neuroticism	0.10	0.12	**0.** **23** **	0.08	**0.** **20** **	**0.** **13** *
Volatility	0.11	0.10	**0.** **18** **	0.08	**0.** **16** *	0.10
Withdrawal	0.07	0.11	**0.** **25** ***	0.04	**0.** **20** **	**0.** **16** *

* *p* ≤ 0.05.

** *p* ≤ 0.01.

*** *p* ≤ 0.001.

Multiple linear regression models were run in which all Big Five traits (or all 10 facets) are treated as simultaneous predictors of each emotional state. If the emotional state was reaction‐based, then the equivalent baseline emotional state was also included as a predictor in the model. These models were run to quantify the unique predictive effect of each Big Five trait or sub‐trait on each emotion measure whilst controlling for shared variance between traits. The full results of regression models can be found in the Supporting Information (Tables S4–, S7).

#### 3.3.1 Openness to Experience, Intellect, & Openness

The hypotheses that Openness to Experience and/or Intellect would have a relationship with Disgust were not supported. Openness to Experience had a non‐significant correlation with Baseline Disgust (*p* = 0.45) and Reaction Disgust (*p* = 0.81). Similarly, Intellect also had a non‐significant correlation with Baseline Disgust (*p* = 0.25) and Reaction Disgust (*p* = 0.76).

The hypotheses that Openness to Experience and/or Openness would have a positive relationship with Joy were not supported. Openness to Experience had a non‐significant correlation with Baseline Joy (*p* = 0.67) and Reaction Joy (*p* = 0.92). Similarly, Openness had a non‐significant correlation with Baseline Joy (*p* = 0.69) and Reaction Joy (*p* = 0.91).

Two significant but non‐hypothesised state‐trait correlations were identified. Firstly, Openness had a small positive correlation with Baseline Sadness (*p* = 0.02). Secondly, Intellect had a small negative correlation with Reaction Suprise (*p* = 0.05). However, only the relationship between Intellect and Reaction Surprise was significant in regression analyses (*p* ≤ 0.01).

#### 3.3.2 Conscientiousness, Industriousness, & Orderliness

The hypotheses that Conscientiousness and/or Industriousness would have a negative relationship with Disgust were partially supported. Conscientiousness had a small negative correlation with Baseline Disgust (*p* ≤ 0.01) and had a non‐significant correlation with Reaction Disgust (*p* = 0.98). However, this relationship was not significant after controlling for covariance between the Big Five traits (*p* = 0.66). Industriousness had a small negative correlation with Baseline Disgust (*p* ≤ 0.01) and had a non‐significant correlation with Reaction Disgust (*p* = 0.69). The relationship between Industriousness and Baseline Disgust was not significant in regression modeling (*p* = 0.55).

The hypotheses that Conscientiousness will have a negative relationship with Anger, Fear, and Sadness were partially supported. Conscientiousness had a small negative correlation with Baseline Anger (*p* = 0.03) and had a non‐significant correlation with Reaction Anger (*p* = 0.12). Conscientiousness had a small negative correlation with Baseline Fear (*p* = 0.02) and had a non‐significant correlation with Reaction Fear (*p* = 0.40). Conscientiousness had a large negative correlation with Baseline Sadness (*p* ≤ 0.001) and had a non‐significant correlation with Reaction Sadness (*p* = 0.63). Follow‐up regression models found that only the relationship between Baseline Sadness and Conscientiousness was significant after controlling for the other Big Five traits (*p* ≤ 0.001).

Sub‐trait analysis found that Orderliness had a moderate negative correlation with Baseline Sadness (*p* ≤ 0.01), and Orderliness also predicted Baseline Sadness (*p* = 0.03) after controlling for shared variance between each sub‐trait. Additionally, Industriousness had a moderate negative correlation with Baseline Anger (*p* ≤ 0.01), a small negative correlation with Baseline Disgust (*p* ≤ 0.01), a moderate negative correlation with Baseline Fear (*p* ≤ 0.001), and a large negative correlation with Baseline Sadness (*p* ≤ 0.001). However, these relationships were not significant after controlling for shared variance between each sub‐trait. Sub‐trait analysis also found that both Orderliness had a moderate negative correlation with Reaction Joy (*p* ≤ 0.001) whereas Industriousness had a small negative correlation with the same emotion (*p* = 0.04), neither of which were hypothesised. However, only the relationship between Orderliness and Reaction Joy was significant in the regression analysis (*p* ≤ 0.01).

#### 3.3.3 Extraversion, Assertiveness, & Enthusiasm

The hypotheses that Extraversion would have a positive relationship with Joy were mostly supported. Extraversion had a moderate positive correlation with Baseline Joy (*p* ≤ 0.001) and Reaction Joy (*p* ≤ 0.01). Enthusiasm had a moderate positive correlation with Baseline Joy (*p* ≤ 0.001) and had a non‐significant correlation with Reaction Joy (*p* = 0.07). The relationship between Extraversion and both Baseline (*p* ≤ 0.001) and Reaction (*p* ≤ 0.01) Joy was statistically significant after controlling for each Big Five trait (and Baseline Joy when predicting Reaction Joy). The relationship between Enthusiasm and Baseline Joy was also significant after regression modeling (*p* ≤ 0.001).

The hypotheses that Extraversion and Assertiveness would have a negative relationship with Fear whereas Enthusiasm would have a positive relationship with Fear were partially supported. Extraversion had non‐significant correlations with Baseline Fear (*p* = 0.10) and Reaction Fear (*p* = 0.08) and Extraversion positively predicted Fear in the regression model (*p* ≤ 0.01). Assertiveness had a small negative correlation with Baseline Fear (*p* = 0.03) and had a non‐significant correlation with Reaction Fear (*p* = 0.86). Enthusiasm had a non‐significant correlation with Baseline Fear (*p* = 0.67) and a small positive correlation with Reaction Fear (*p* ≤ 0.01). However, none of these relationships were significant in the regression analysis.

Several non‐hypothesised significant correlations were also identified. Extraversion had a small positive correlation with both Reaction Anger (*p* = 0.02) and Disgust (*p* = 0.03). Both relationships were significant in the regression analysis (both *p* = 0.02). Enthusiasm had a small positive correlation with both Reaction Anger (*p* = 0.04), Disgust (*p* = 0.04), and Sadness (*p* = 0.04). However, Enthusiasm did not significantly predict any of these emotions in the regression analysis.

#### 3.3.4 Agreeableness, Compassion, & Politeness

The hypotheses that Agreeableness, Compassion, and Politeness would have a negative relationship with Anger were largely not supported. Agreeableness had non‐significant correlations with Baseline Anger (*p* = 0.46) and Reaction Anger (*p* = 0.27). The regression analysis did show, however, that Agreeableness positively predicted Reaction Anger (*p* = 0.05). Compassion had non‐significant correlations with Baseline Anger (*p* = 0.41) and Reaction Anger (*p* = 0.06). Politeness had a small negative correlation with Baseline Anger (*p* = 0.02) and had a non‐significant correlation with Reaction Anger (*p* = 0.91). None of these relationships were found to be significant in regression analysis.

Several non‐hypothesised significant correlations were also identified. Agreeableness had a small positive correlation with Reaction Disgust (*p* = 0.02), a moderate positive correlation with Reaction Fear (*p* ≤ 0.001), and a small positive correlation with Reaction Sadness (*p* ≤ 0.01). The regression analysis showed that the relationships with Reaction Disgust (*p* = 0.02), Fear (*p* ≤ 0.001), and Sadness (*p* ≤ 0.01) were all significant after controlling for the other Big Five traits. In terms of sub‐traits, Compassion had a moderate positive correlation with Reaction Disgust (*p* ≤ 0.01), a large positive correlation with Reaction Fear (*p* ≤ 0.001), and a moderate correlation with Reaction Sadness (*p* ≤ 0.001). However, only the relationship with Reaction Fear was significant in the regression analysis (p ≤ 0.01). Politeness had small negative correlations with Reaction Joy (*p* = 0.05) and Baseline Disgust (*p* = 0.04). However, only the relationship with Baseline Disgust was significant in the regression analysis (*p* = 0.02)

#### 3.3.5 Neuroticism, Volatility, & Withdrawal

The hypotheses that Neuroticism would have a positive relationship with Fear were supported. Neuroticism had a large positive correlation with Baseline Fear (*p* ≤ 0.001) and had a moderate positive correlation with Reaction Fear (*p* ≤ 0.001). Regression analyses supported these findings for both Baseline (*p* ≤ 0.001) and Reaction Fear (*p* ≤ 0.001). Withdrawal had a very large positive correlation with Baseline Fear (*p* ≤ 0.001) and had a moderate positive correlation with Reaction Fear (*p* ≤ 0.001). Regression analysis showed that only Withdrawal's relationship with Baseline Fear is robust (*p* ≤ 0.001). Volatility had a moderate positive correlation with Baseline Fear (*p* ≤ 0.001) and Reaction Fear (*p* ≤ 0.01). Neither relationship was identified as significant in regression analyses.

The hypotheses that Neuroticism would have a positive relationship with Sadness were supported. Neuroticism had a moderate positive correlation with both Baseline Sadness (*p* ≤ 0.001) and Reaction Sadness (*p* ≤ 0.01). Neuroticism's relationships with both Baseline (*p* ≤ 0.01) and Reaction Sadness (*p* = 0.01) were found to be robust. Volatility had a moderate positive correlation with both Baseline Sadness (*p* ≤ 0.01) and Reaction Sadness (*p* = 0.03). However, neither relationship was identified as significant in regression analyses. Withdrawal had a moderate positive correlation with Baseline Sadness (*p* ≤ 0.01) that was also robust in the regression analysis (*p* = 0.02).

Several non‐hypothesised significant correlations were also identified. Baseline Sadness had moderate positive correlations with Neuroticism (*p* ≤ 0.001) and Volatility (p ≤ 0.01), and a large positive relationship with Withdrawal (*p* ≤ 0.001). Baseline Sadness was also significantly predicted by Neuroticism (*p* ≤ 0.01) and Withdrawal (*p* = 0.02) in the regression analysis. Reaction Sadness had small positive correlations with Neuroticism (*p* ≤ 0.01), Volatility (*p =* 0.03), and Withdrawal (*p* ≤ 0.01). However, only Neuroticism predicted Reaction Sadness in the regression analysis (*p* = 0.01). Finally, Reaction Surprise had a small positive correlation with Withdrawal (*p* = 0.03) that was also identified in the regression analysis (*p* = 0.02).

## 4 Discussion

The relationships between FFM personality traits and basic emotional states have not been extensively investigated. Most research has focused on (i) dimensional attributes of affect, (ii) Extraversion and Neuroticism's relationship with emotional experiences, and (iii) the Big Five level of the trait hierarchy. This exploratory study aimed to broaden the scope of personality‐emotion research. This was realized by quantifying the relationships between FFM personality traits, their sub‐trait traits, and basic emotional experiences before and after participants watched a set of emotionally provocative video clips. This study investigated the research question: What are the relationships between FFM personality traits and basic emotional states? This section discusses the most significant results for each FFM trait in relation to the research question, hypotheses (see Table S1), and relevant literature. Noteworthy findings that were not hypothesized but provide context to the empirical literature on personality, emotions, and their relationships are also discussed.

### 4.1 Openness to Experience

Openness to Experience had no relationship with Disgust. This result contradicts research reporting a negative state–trait relationship (Druschel and Sherman 1999) but is consistent with other research findings (Giampietro et al. 2019). Consequently, this finding does not support the claim that Openness to Experience partially explains individual differences in Disgust sensitivity. Research has found that people are more likely to be lower in Openness to Experience within countries that have higher levels of disease prevalence (Schaller and Murray 2008). The results here suggest that this relationship may be due to other factors outside Disgust sensitivity. For example, Openness to Experience may predict greater awareness of the risks of infectious diseases, which thereby predicts greater preventive action and thus reduces disease transmission (Stadler et al. 2020).

Openness to Experience had no relationship with Joy. It was expected that people higher in Openness to Experience would experience more Joy. This expectation was based on past research findings that reported a positive correlation between Openness to Experience and positive affect or neurobiological systems that underlie positive affect (Anglim et al. 2020; Marengo et al. 2021). The results of this study suggest that this relationship between Openness to Experience and positive affect may be mediated by non‐Joy‐based aspects of positive affect, such as interest, attentiveness, or alertness. Such aspects overlap considerably with cognitive processes. Since Openness to Experience is a cognitive‐based trait, this overlap may explain previous research findings. Further research that maps Openness to Experience to individual facets of positive affect is recommended.

There were also some noteworthy findings that were not hypothesized. The only significant relationship found between Openness to Experience, and basic emotional states was that higher levels of Openness to Experience were associated with lower levels of Reaction Surprise (*β* = −0.243). This relationship is likely due to its sub‐trait Intellect, which negatively predicted Reaction Sadness in both correlation and regression analysis. This effect may have been mediated by cognitive processes. Intellect's function has been defined as reflecting “detection of logic or causal pattern in abstract and semantic information” (DeYoung 2015a, 42). Consequently, people higher in Intellect may have been more likely to acquire relevant information from the videos and predict the outcome, thereby reducing the amount of Surprise they experienced. This could be tested in future studies by including measures of cognitive engagement or attention (e.g., eye tracking).

A positive relationship was found between Openness and Baseline Sadness that is consistent with findings showing that facets of Openness are positively associated with negative affect disorder diagnoses (e.g., Major Depression Disorder; Walton et al. 2018). A higher likelihood of experiencing Sadness may interact with the relationship between Openness facets and such disorders. However, this effect was not replicated in the regression analyses. Future research can aim to evaluate this potential relationship and quantify any potential interaction between Openness, negative affect states, and affective disorders.

### 4.2 Conscientiousness

Conscientious people experience less negative affect in their daily lives, but they are not less sensitive to such states. Conscientiousness negatively correlated with Baseline Anger, Disgust, Fear, and Sadness, which is consistent with findings from meta‐analytical research and with several of this study's hypotheses (Anglim et al. 2020; DeNeve and Cooper 1998; Fayard et al. 2012; Joshanloo 2019). However, regression analyses showed that Conscientiousness was only able to uniquely predict Baseline Sadness after including each of the Big Five as simultaneous predictors.

Research on the existence of meta‐traits that explain coordination between the Big Five traits argues that Conscientiousness, Agreeableness, and Emotional Stability (Neuroticism reversed) form the meta‐trait Stability (DeYoung 2015a). One defining aspect of Stability is that people high on this trait are resistant to life experiences that can potentially create psychological entropy. While no relationship was found here between Conscientiousness and Agreeableness, a negative correlation was found between this trait and Neuroticism. Consequently, people high in Conscientiousness may be less likely to experience negative affect due to being higher on emotional stability. Future research that investigates the interplay between Conscientiousness, Neuroticism, and Agreeableness in relation to negative emotional experiences may provide support for the existence of the Stability meta‐trait.

These findings also suggest that people high in Conscientiousness may be acting in unique ways that make them less likely to experience Sadness. In support of this explanation, previous research studies have shown that people high in Conscientiousness (i) use more productive coping styles that reduce the likelihood of experiencing long and intense negative affect (Javaras et al. 2012) and (ii) are more sensitive to experiencing guilt, and this motivates Conscientious individuals to avoid impulsive behavior that may cause negative emotion (e.g., cheating on a partner, not studying for an exam; Fayard et al. 2012). In particular, the Orderliness sub‐trait of Conscientiousness was found to significantly predict reduced experience of Sadness but slightly higher reactivity to the same emotion. This finding suggests that how people structure their environment may be a key shield from experiencing Sadness, which may represent a significant motivator for people high in Orderliness if they are sensitive to this emotion.

Conscientious people are less likely to experience Disgust. However, Conscientiousness was not a unique predictor of Disgust after controlling for other Big Five traits. This contradicts the claim that conscientious behavior is motivated by a higher level of Disgust sensitivity (Druschel and Sherman 1999; Giampietro et al. 2019; Tybur et al. 2009, 2011; Tybur and de Vries 2013). The differences in findings between past studies and this study may be a consequence of differences in how Disgust was measured. Previous studies asked participants to self‐report how disgusting they would find hypothetical situations (i.e., trait Disgust). In contrast, this study asked participants to self‐report their current level of Disgust in response to real emotional stimuli (i.e., state Disgust). People high in Conscientiousness may overestimate their level of trait Disgust, as research has shown that such individuals experience higher levels of guilt and employ harsher moral judgments (Fayard et al. 2012). It is recommended that future research studies investigating this relationship use both trait and state Disgust measures.

The overall lack of a relationship between Conscientiousness and reaction to negative affect states is consistent with prior research that showed people high in Conscientiousness are more likely to recover from negative affect, but they are neither more nor less reactive to negative affect (Javaras et al. 2012).

### 4.3 Extraversion

Extraverts are more likely to experience Joy. In line with the study's hypothesis, Extraversion had a positive relationship with both Baseline (*r*_s_ = 0.23) and Reaction Joy (r_s_ = 0.18). These relationships were robust when controlling for shared variance between the Big Five. Overall, this result supports the claim that Extraversion is temperamentally related to Joy (Anglim et al. 2020; DeNeve and Cooper 1998; Joshanloo 2019; Lucas and Baird 2004; Lucas et al. 2008; Smillie et al. 2015; Watson and Clark 1992).

Previous research has suggested that Assertiveness and Enthusiasm are distinctly related to Joy (DeYoung 2015a; Smillie et al. 2013, 2015), such that Assertiveness is related to goal‐based positive affect (associated with Baseline Joy) whereas Enthusiasm is related to reward‐based positive affect (associated with Reaction Joy). The results here do support the claim that these sub‐traits are distinctly related to Joy, but not in the expected direction. Enthusiasm was associated with Baseline Joy whereas Assertiveness was associated with Reaction Joy, and these associations were robust in regression analyses. The findings that Enthusiasm makes one more likely to experience Joy may reflect the general positive nature of this sub‐trait (and Extraversion in general). In contrast, the finding that Assertiveness makes one more sensitive to Joy may be a key factor in explaining why people high in this trait can be so goal‐driven, as the pursuit of rewards is likely to bring higher levels of Joy.

### 4.4 Agreeableness

Agreeableness is an important predictor of emotional experiences. Agreeableness significantly correlated with three emotional states (Reaction Anger, Disgust, and Fear), with only Neuroticism having a higher number of significant correlations (4). Out of the 12 regression models computed, 8 of those models found Agreeableness to be a significant predictor of emotional experiences after controlling for other variables. This finding generally supports the conceptualisation of Agreeableness found in scales that use a large amount of affect content to measure Agreeableness (e.g., 43.70% in the Mini‐Marker) compared to scales with a low amount of affective content (e.g., 10.50% in the NEO Five‐Factor Inventory; Zillig et al. 2002). These latter scales may be significantly underestimating Agreeableness' affective dimensions, thereby impairing their ability to identify Agreeableness' relationships with life outcomes or psychological phenomena that are predicated on the trait's affective nature.

It was hypothesized that Agreeableness would negatively correlate with Anger. However, the relationship found between Agreeableness and Anger in this study is slightly complex. No significant correlation was found between Agreeableness and either instance of Anger, but Agreeableness did positively and uniquely predict Reaction Anger in regression analyses. While this may reflect a true state–trait relationship, there are two reasons to be cautious of this finding. Firstly, Agreeableness barely met the statistically significant value threshold (*p* = 0.049 when reporting to three decimal places). Given the exploratory nature of the study, this finding may be a statistical artifact of the high degree of testing done in this study. Secondly, previous studies have indicated that Agreeable people are less sensitive and less likely to experience Anger (Bresin et al. 2012; Özyeşil 2012; Pfeiler et al. 2018). While other research has suggested that Agreeableness is associated with being less likely to express rather than experience Anger, it has not been claimed that Agreeableness should be positively associated with Anger. Future research may need to explore Agreeableness relationship with Anger across multiple contexts and measures to enable a full understanding of their relationshi

Agreeable people are more likely to be sensitive to Fear, Disgust, and Sadness. Agreeableness had a positive relationship with the Reaction experience of each state, which was not hypothesized. However, this result supports findings that people high in narrow traits related to Agreeableness (e.g., positive relationships with others; see Schmutte and Ryff 1997) are more likely to be sensitive to negative affect (Schaefer et al. 2013). At the same time, this result contradicts meta‐analytical research that reported a negative relationship between Agreeableness and negative affect (Anglim et al. 2020). This contradiction may be a result of the differences in how dimensional and discrete approaches classify emotions. Given that dimensional approaches cluster separate emotions together, if Agreeableness has differential relationships with different negative emotional states, this may cause inconsistency in results. It may be the case that Agreeableness can negatively correlate with negative affect but have positive correlations with discrete negative affect states.

### 4.5 Neuroticism

People high in Neuroticism are more likely to experience Fear and Sadness and are more sensitive to such states. Neuroticism positively and uniquely predicted both emotional states pre‐video and post‐video. These results support the study's hypotheses and are consistent with the claim that Neuroticism is directly (i.e., “temperamentally”) related to negative emotional states and that Neuroticism is a “Negative Affect” trait.

A non‐hypothesized finding was that people high in Neuroticism are more likely to experience Anger but are not more sensitive to this emotion. Neuroticism had a positive relationship with Baseline Anger but had no relationship with Reaction Anger. This finding contradicts past research which reported that Neuroticism had a positive relationship with Reaction Anger but had no relationship with Baseline Anger (Pfeiler et al. 2018). This contradiction may be a result of differences in methodology. This study induced Reaction Anger using a non‐goal‐based task (video stimuli), whereas in the study conducted by Pfeiler et al. (2018), Reaction Anger was induced by having a confederate interrupt their participants during a goal‐based task. Conceptually, Neuroticism is argued to be associated with higher negative affect when one's goals are challenged (DeYoung 2015a). If this is the case, this may be the cause of the different state–trait relationships found for Reaction Anger. This explanation could be tested in future studies by evaluating whether the type of emotion induction (goal‐based vs. non‐goal‐based) makes people high in Neuroticism more sensitive to negative emotion.

The differences in Baseline Anger may be due to the different FFM scales used. This study used the 100‐item BFAS scale, whereas the study conducted by Pfeiler et al. (2018) used the 10‐item Big Five inventory. Psychometric researchers recommend using scales that contain at least 8–12 items per Big Five trait (Anglim and O'Connor 2019). Scales with fewer items are more likely to suffer from low internal consistency and poor external reliability. These points are acknowledged by Pfeiler et al. (2018) in their study. Additionally, the non‐significant correlation between Neuroticism and Baseline Anger reported in their study was positive (*r* = 0.12). If their study were replicated with a more extensive measure of Neuroticism, then the outcome may be consistent with the results found here.

### 4.6 On the Granularity of the Approach

This study evaluated personality and emotions at different levels of granularity. Personality was measured at relatively high levels of granularity (the Big Five and their sub‐traits), whereas emotion was measured at a relatively low level of granularity (basic emotional states). This begs the question, why not measure both phenomena at a similar level? There were two reasons we opted for this approach. Firstly, the sub‐traits have been proposed as a potential solution to the bandwidth‐fidelity dilemma, in that they enable a higher degree of fidelity predictions whilst conserving bandwidth. We were interested in evaluating this claim about state–trait research.

Secondly, state–trait research has tended to focus on higher‐level constructs such as Arousal or Valence. While this is a valid approach, reliance on these constructs makes it difficult to understand how specific emotional experiences relate to personality. It was posited that measuring emotions at a discrete level would provide valuable and more specific information on state–trait relationships. Additionally, there is valuable insight in knowing whether a person is prone to specific emotional states. People who experience high levels of Anger are more likely to be verbally and physically aggressive, which can have devastating life outcomes for themselves and other people (Ekman 2004). People who experience high levels of Sadness are more likely to experience clinical and affective disorders (Leventhal 2008). By understanding whether broad personality traits like the Big Five and their sub‐traits relate to specific emotional experiences, this will enable further investigation on potential pathways from psychological phenomena to life outcomes.

### 4.7 Limitations

This study focused on FFM personality traits' relationships with basic emotional states, selected for their reliability and foundational role in broader emotional experiences. However, this approach does not capture variability within basic emotions, which consist of interrelated states. For example, Disgust can be decomposed into different types (Moral, Sexual, Pathogen; Tybur et al. 2009). Focusing on high‐level representations may oversimplify their relationships with FFM traits. Future research could address this by incorporating measures of arousal and valence alongside basic emotions.

A key methodological decision was using a global approach to analyze FFM traits and reaction‐based emotional states. While this approach has advantages (see Design subsection), averaging emotional experiences across stimuli may obscure important variation. A larger sample and multi‐level modeling could mitigate this limitation by enabling both local and global analyses.

The sample size collected in this study is capable of reliably detecting relationships with moderate and large effect sizes but is unable to detect small and very small effect sizes (Funder and Ozer 2019). This is a limitation of the study as small and very small effect sizes can have considerable influence on a person's psychological experiences over the long term. The power of such influence is greater in the case of ubiquitous psychological phenomena such as personality traits and emotional experiences. It is not a trivial fact if a person high in a trait or sub‐trait is more likely to experience Joy on a day‐to‐day basis over months, years, and decades of his or her life. Additionally, both small and very small effect sizes are likely to be more abundant in the real world (Funder and Ozer 2019). Consequently, while this study clarifies moderate and large state–trait relationships, it also highlights the need for research with larger samples to detect small but meaningful effects.

Many analyses were conducted in this study without post hoc corrections. This decision was made because the authors aimed to explore state–trait relationships that are worth further investigation and minimize the likelihood of making Type II errors. However, this decision means that the analyses were more likely to make Type I errors. Consequently, any significant results identified here should be the subject of replication attempts.

Similarly, several variables were not normally distributed. Although multiple linear regression is generally robust to normality deviations in large samples (Schmidt and Finan 2018; Lumley et al. 2002), some models exhibited mild violations of linearity or heteroscedasticity. While these were not severe enough to invalidate findings, they highlight a need for further research that explores alternative modeling approaches, such as robust regression or generalized additive models, to account for potential non‐linear relationships.

Finally, this study did not examine sex or gender differences in the associations between personality traits and emotional experiences. While such differences can provide valuable insights, our primary aim was to assess overall trait‐emotion relationships without introducing additional complexity that could detract from the clarity of the findings. Moreover, recent findings suggest Big Five trait‐outcome associations are largely consistent across gender identities (Soto 2021), indicating that gender may not be a critical moderator in this context. Future studies could examine whether this pattern holds for trait–state relationships and whether similar consistency is observed across sexes.

## 5 Conclusion

Much of the existing research on personality traits and emotional states has overlooked the potential contributions of basic emotional experiences, the personality traits of Openness to Experience, Conscientiousness, and Agreeableness, and sub‐traits in understanding state–trait relationships. This study demonstrates the value of each aspect. Firstly, the findings here demonstrate that basic emotions enable one to identify more specific state–trait relationships than can be identified by using a dimensional approach alone. Secondly, Conscientiousness uniquely predicted reduced experiences of Sadness, whereas Agreeableness uniquely predicted higher sensitivity to several negative emotional states. This finding contradicts the claim that the traits Conscientiousness and Agreeableness are cognitive‐behavioral traits that are not significantly related to emotional experiences. Instead, the results here suggest that both Conscientiousness and Agreeableness have relationships with emotional experiences that are more nuanced and complex than previous research has assumed. In contrast, the findings here suggest that Openness to Experience uniquely predicts reduced experiences of Surprise. Thirdly, the sub‐traits were able to provide more specific information on what aspects of the trait were associated with the emotional state (e.g., Orderliness with Sadness and Intellect with Surprise). The study supports the conclusion that the sub‐traits are valuable constructs for deepening one's understanding of the Big Five traits.

## Author Contributions

**Ryan Donovan:** conceptualisation, data collection, data curation, formal analysis, methodology, investigation, writing – original draft preparation, writing – review and editing. **Aoife Johnson:** writing – review and editing, conceptualisation, investigation, methodology, supervision. **Aine de Roiste:** writing – review and editing, supervision. **Ruairi O'Reilly:** conceptualisation, methodology, investigation, writing – original draft preparation, writing – review and editing, supervision.

## Conflicts of Interest

The authors declare no conflicts of interest.

## Supporting information


**Table S1.** Summary of hypotheses broken down per Big Five trait and basic emotional state.

**Table S2.** Pairwise correlation between Big Five personality traits, sub‐traits, and emotional states (baseline and reaction).

Data S1.

**Table S4.** The results of regression models where each Big Five trait was entered as simultaneous predictors for each baseline emotion.

**Table S5.** The results of regression models where each Big Five trait was entered as simultaneous predictors for each reaction emotion.

**Table S6.** The results of regression models where each sub‐trait was entered as simultaneous predictors for each baseline emotion.

**Table S7.** The results of regression models where each sub‐trait was entered as simultaneous predictors for each reaction emotion.
