# Supplementary material for: Investigating the Relationships Between Basic Emotions and the Big Five Personality Traits and Their Sub‐Traits
Source: J Pers. 2025 May 15;94(2):237–51. doi: 10.1111/jopy.13027 (PMC12988340; doi:10.1111/jopy.13027)
Supplement: Supplementary file 1 — Table S1. Summary of hypotheses broken down per Big Five trait and basic emotional state. [file JOPY-94-237-s004.docx]

**Table S1**

*Summary of hypotheses broken down per Big Five trait and basic emotional state.*

| Trait | Basic Emotion | Hypothesis (H_1_) |
| --- | --- | --- |
| Openness to Experience | Disgust | Openness to Experience will have a negative relationship with Disgust |
|  | Joy | Openness to Experience will have a positive relationship with Joy |
| Conscientiousness | Anger | Conscientiousness will have a negative relationship with Anger |
|  | Fear | Conscientiousness will have a negative relationship with Fear |
|  | Sadness | Conscientiousness will have a negative relationship with Sadness |
|  | Disgust | Conscientiousness will have a positive relationship with Sadness |
| Extraversion | Joy | Extraversion will have a positive relationship with Joy |
| Agreeableness | Anger | Agreeableness will have a negative relationship with Anger |
| Neuroticism | Fear | Neuroticism will have a negative relationship with Fear |
|  | Sadness | Neuroticism will have a negative relationship with Sadness |
