# Supplementary material for: Investigating the Relationships Between Basic Emotions and the Big Five Personality Traits and Their Sub‐Traits
Source: J Pers. 2025 May 15;94(2):237–51. doi: 10.1111/jopy.13027 (PMC12988340; doi:10.1111/jopy.13027)
Supplement: Supplementary file 2 — Table S2. Pairwise correlation between Big Five personality traits, sub‐traits, and emotional states (baseline and reaction). [file JOPY-94-237-s001.docx]

**Table S2**

*Pairwise correlation between Big Five personality traits, sub-traits, and emotional states (baseline and reaction).*

|  | ***1*** | ***2*** | ***3*** | ***4*** | ***5*** | ***6*** | ***7*** | ***8*** | ***9*** | ***10*** | ***11*** | ***12*** | ***13*** | ***14*** | ***15*** | ***16*** | ***17*** | ***18*** | ***19*** | ***20*** | ***21*** | ***22*** | ***23*** | ***24*** | ***25*** | ***26*** |
| --- | --- | --- | --- | --- | --- | --- | --- | --- | --- | --- | --- | --- | --- | --- | --- | --- | --- | --- | --- | --- | --- | --- | --- | --- | --- | --- |
| **1:OpenE** |  |  |  |  |  |  |  |  |  |  |  |  |  |  |  |  |  |  |  |  |  |  |  |  |  |  |
| **2:Int** | **.77*** |  |  |  |  |  |  |  |  |  |  |  |  |  |  |  |  |  |  |  |  |  |  |  |  |  |
| **3:Open** | **.80*** | **.26*** |  |  |  |  |  |  |  |  |  |  |  |  |  |  |  |  |  |  |  |  |  |  |  |  |
| **4:Con** | .10 | **.28*** | -.11 |  |  |  |  |  |  |  |  |  |  |  |  |  |  |  |  |  |  |  |  |  |  |  |
| **5:Ind** | .10 | **.34*** | **-.15*** | **.87*** |  |  |  |  |  |  |  |  |  |  |  |  |  |  |  |  |  |  |  |  |  |  |
| **6:Ord** | .09 | **.17*** | -.05 | **.86*** | **.51*** |  |  |  |  |  |  |  |  |  |  |  |  |  |  |  |  |  |  |  |  |  |
| **7:Ext** | **.26*** | **.36*** | .12 | **.24*** | **.30*** | **.15*** |  |  |  |  |  |  |  |  |  |  |  |  |  |  |  |  |  |  |  |  |
| **8:Asst** | **.33*** | **.49*** | .11 | **.30*** | **.36*** | **.18*** | **.87*** |  |  |  |  |  |  |  |  |  |  |  |  |  |  |  |  |  |  |  |
| **9:Enth** | .11 | .11 | .12 | .08 | .13 | .03 | **.81*** | **.45*** |  |  |  |  |  |  |  |  |  |  |  |  |  |  |  |  |  |  |
| **10:Agreablness** | **.25*** | .02 | **.37*** | .04 | .01 | .07 | .13 | -.05 | **.33*** |  |  |  |  |  |  |  |  |  |  |  |  |  |  |  |  |  |
| **11:Comp** | **.37*** | **.15*** | **.44*** | -.06 | -.11 | .03 | **.32*** | .12 | **.48*** | **.83*** |  |  |  |  |  |  |  |  |  |  |  |  |  |  |  |  |
| **12:Pol** | .03 | -.13 | **.15*** | **.15*** | **.14*** | .12 | **-.17*** | **-.26*** | .01 | **.78*** | **.35*** |  |  |  |  |  |  |  |  |  |  |  |  |  |  |  |
| **13:Neu** | -.10 | **-.28*** | .09 | **-.34*** | **-.52*** | -.09 | **-.25*** | **-.31*** | -.11 | .06 | **.15*** | -.09 |  |  |  |  |  |  |  |  |  |  |  |  |  |  |
| **14:Vol** | -.07 | **-.22*** | .09 | **-.28*** | **-.42*** | -.08 | -.11 | **-.17*** | -.03 | -.07 | .08 | **-.25*** | **.92*** |  |  |  |  |  |  |  |  |  |  |  |  |  |
| **15:With** | -.11 | **-.32*** | .10 | **-.35*** | **-.55*** | -.08 | **-.37*** | **-.44*** | **-.19*** | **.18*** | **.19*** | .10 | **.89*** | **.67*** |  |  |  |  |  |  |  |  |  |  |  |  |
| **16:AnR** | -.09 | -.05 | -.06 | .11 | .07 | .12 | **.17*** | .12 | **.15*** | .08 | .13 | -.01 | .10 | .11 | .07 |  |  |  |  |  |  |  |  |  |  |  |
| **17:DisR** | -.02 | -.02 | .02 | .00 | -.03 | .04 | **.15*** | .08 | **.15*** | **.17*** | **.20*** | .06 | .12 | .10 | .11 | **.74*** |  |  |  |  |  |  |  |  |  |  |
| **18:FeaR** | -.01 | -.07 | .07 | -.06 | -.10 | .00 | .12 | .01 | **.18*** | **.27*** | **.32*** | .11 | .23 | **.18*** | **.25*** | **.58*** | **.63*** |  |  |  |  |  |  |  |  |  |
| **19:JoyR** | -.01 | .01 | -.01 | **-.23*** | **-.15*** | **-.24*** | **.18*** | .15 | .13 | -.08 | -.02 | **-.14*** | .08 | .09 | .04 | .16* | .11 | **.15*** |  |  |  |  |  |  |  |  |
| **20:SadR** | .02 | -.02 | .07 | -.03 | -.05 | -.01 | .13 | .05 | **.15*** | **.19*** | **.24*** | .04 | **.20*** | **.16*** | **.20*** | **.59*** | **.56*** | **.66*** | **.30*** |  |  |  |  |  |  |  |
|  | ***1*** | ***2*** | ***3*** | ***4*** | ***5*** | ***6*** | ***7*** | ***8*** | ***9*** | ***10*** | ***11*** | ***12*** | ***13*** | ***14*** | ***15*** | ***16*** | ***17*** | ***18*** | ***19*** | ***20*** | ***21*** | ***22*** | ***23*** | ***24*** | ***25*** | ***26*** |
| **21:SurR** | -.07 | **-.14*** | .03 | .01 | -.02 | .00 | .08 | .03 | .09 | .10 | .12 | .09 | .13 | .09 | **.16*** | **.51*** | **.53*** | **.57*** | **.30*** | **.44*** |  |  |  |  |  |  |
| **22:AnB** | -.06 | -.07 | -.01 | **-.15*** | **-.22*** | -.04 | .05 | .04 | .02 | -.05 | .06 | **-.17*** | **.27*** | **.26*** | **.26*** | **.22*** | **.16*** | **.23*** | .06 | **.23*** | .11 |  |  |  |  |  |
| **23:DisB** | -.05 | -.08 | .01 | **-.18*** | -**.19*** | -.11 | .02 | -.03 | .05 | -.06 | .04 | **-.15*** | .03 | .07 | .01 | .08 | .05 | .03 | -.02 | .06 | .03 | **.38*** |  |  |  |  |
| **24:FeaB** | -.01 | -.10 | .06 | **-.16*** | **-.25*** | -.02 | -.12 | **-.16*** | -.03 | .13 | .11 | .11 | **.31*** | .18* | **.40*** | **.18*** | .11 | **.23*** | **.15*** | .18* | **.14*** | **.23*** | **.17*** |  |  |  |
| **25:JoyB** | -.03 | .03 | -.03 | .08 | .08 | .05 | **.23*** | .13 | **.28*** | .06 | .08 | .01 | -.12 | -.11 | -.12 | .23* | **.23*** | **.20*** | .14 | **.20*** | **.16*** | **.14*** | **.15*** | .13 |  |  |
| **26:SadB** | .01 | -.13 | **.17*** | **-.30*** | **-.32*** | **-.21*** | -.08 | -.13 | -.02 | .05 | .11 | -.06 | **.29*** | **.22*** | **.32*** | **.19*** | **.19*** | **.26*** | **.14*** | **.31*** | **.21*** | **.45*** | **.25*** | **.35*** | .08 |  |
| **27:SurB** | .10 | .09 | .07 | .00 | .00 | .00 | .08 | .06 | .07 | .00 | .05 | -.06 | -.10 | -.09 | -.09 | .13 | **.15*** | .12 | .11 | **.16*** | .12 | .09 | .14 | .21* | **.46*** | **.21*** |

**Note: *** indicates a correlation that is statistically significant ***p < .05***. To view correlation matrix with exact p-values, see the study’s page on OSF:

The following acronyms were used to ensure table met page margins:

**OpenE** = *Openness to Experience*; **Open** = *Openness*; **Int** = *Intellect*; ***Con*** = *Conscientiousness*; ***Ind*** = *Industriousness*; ***Ord*** = *Orderliness*; ***Ext*** = *Extraversion*; ***Asst*** = *Assertiveness*; ***Enth*** = *Enthusiasm*; ***Ag*** = *Agreeableness*; ***Comp*** = *Compassion*; ***Pol*** = *Politeness*; ***Neu*** = *Neuroticism*; ***With*** = *Withdrawal*; ***Vol*** = *Volatility*; ***AnB*** = *Anger Baseline*; ***AnR*** = *Anger Reaction*; ***DisB*** = *Disgust Baseline*; ***DisR*** = *Disgust Reaction*; ***FeaB*** = *Fear Baseline*; ***FeaR*** = *Fear Reaction*; ***JoyB*** = *Joy Baseline*; ***JoyR*** = *Joy Reaction*; ***SadB*** = *Sadness Baseline*; ***SadR*** = *Sadness Reaction*; ***SurB*** = *Surprise Baseline*; ***SurR*** = *Surprise Reaction*.
