# Supplementary material for: Investigating the Relationships Between Basic Emotions and the Big Five Personality Traits and Their Sub‐Traits
Source: J Pers. 2025 May 15;94(2):237–51. doi: 10.1111/jopy.13027 (PMC12988340; doi:10.1111/jopy.13027)
Supplement: Supplementary file 3 — Data S1. [file JOPY-94-237-s005.docx]

**Supplementary Materials – Study Materials**

The following materials were used in the study titled: "Beyond Affect: Exploring the Basic Emotional Dimensions of The Big Five and their Sub-Traits" that has been submitted to the Journal of Personality.

Contents

[Big Five Aspects Scale 2](#_Toc174960645)

[BFAS Scoring Key: 4](#_Toc174960646)

[Discrete Emotion Questionnaire 6](#_Toc174960647)

[Reaction DEQ 6](#_Toc174960648)

[Baseline DEQ 6](#_Toc174960649)

[Audio Task 7](#_Toc174960650)

[Audio Description 7](#_Toc174960651)

[Audio Prompt 7](#_Toc174960652)

[Cognitive Reflection Test 8](#_Toc174960653)

[Video Description (Table S3) 10](#_Toc174960654)

# Big Five Aspects Scale

Here are a number of characteristics that may or may not describe you. For example, do you agree that you rarely feel blue, compared to most other people? Please fill in the number that best indicates the extent to which you agree or disagree with each statement listed below. Be as honest as possible but rely on your initial feeling and do not think too much about each item.

Please rate each characteristic using the following scale:

1 – Strongly Disagree

2 – Disagree

3 – Neither Agree nor Disagree

4 – Agree

5 – Strongly Agree

1. I rarely feel blue.
2. I am not interested in other people's problems.
3. I carry out my plans.
4. I make friends easily.
5. I am quick to understand things.
6. I get angry easily.
7. I respect authority.
8. I leave my belongings around.
9. I take charge.
10. I enjoy the beauty of nature.
11. I am filled with doubts about things.
12. I feel others' emotions.
13. I waste my time.
14. I am hard to get to know.
15. I have difficulty understanding abstract ideas.
16. I rarely get irritated.
17. I believe that I am better than others.
18. I like order.
19. I have a strong personality.
20. I believe in the importance of art.
21. I feel comfortable with myself.
22. I inquire about others' well-being.
23. I find it difficult to get down to work.
24. I keep others at a distance.
25. I can handle a lot of information.
26. I get upset easily.
27. I hate to seem pushy.
28. I keep things tidy.
29. I lack the talent for influencing people.
30. I love to reflect on things.
31. I feel threatened easily.
32. I can't be bothered with others' needs.
33. I mess things up.
34. I reveal little about myself.
35. I like to solve complex problems.
36. I keep my emotions under control.
37. I take advantage of others.
38. I follow a schedule.
39. I know how to captivate people.
40. I get deeply immersed in music.
41. I rarely feel depressed.
42. I sympathize with others' feelings.
43. I finish what I start.
44. I warm up quickly to others.
45. I avoid philosophical discussions.
46. I change my mood a lot.
47. I avoid imposing my will on others.
48. I am not bothered by messy people.
49. I wait for others to lead the way.
50. I do not like poetry.
51. I worry about things.
52. I am indifferent to the feelings of others.
53. I don't put my mind on the task at hand.
54. I rarely get caught up in the excitement.
55. I avoid difficult reading material.
56. I rarely lose my composure.
57. I rarely put people under pressure.
58. I want everything to be "just right".
59. I see myself as a good leader.
60. I rarely notice the emotional aspects of paintings and pictures.
61. I am easily discouraged.
62. I take no time for others.
63. I get things done quickly.
64. I am not a very enthusiastic person.
65. I have a rich vocabulary.
66. I am a person whose moods go up and down easily.
67. I insult people.
68. I am not bothered by disorder.
69. I can talk others into doing things.
70. I need a creative outlet.
71. I am not embarrassed easily.
72. I take an interest in other people's lives.
73. I always know what I am doing.
74. I show my feelings when I'm happy.
75. I think quickly.
76. I am not easily annoyed.
77. I seek conflict.
78. I dislike routine.
79. I hold back my opinions.
80. I rarely get lost in thought.
81. I become overwhelmed by events.
82. I don't have a soft side.
83. I postpone decisions.
84. I have a lot of fun.
85. I learn things slowly.
86. I get easily agitated.
87. I love a good fight.
88. I see that rules are observed.
89. I am the first to act.
90. I rarely daydream.
91. I am afraid of many things.
92. I like to do things for others.
93. I am easily distracted.
94. I laugh a lot.
95. I formulate ideas clearly.
96. I can be stirred up easily.
97. I am out for my own personal gain.
98. I want every detail taken care of.
99. I do not have an assertive personality.
100. I see the beauty in things that others might not notice.

## BFAS Scoring Key:

Neuroticism

Withdrawal: 1R, 11, 21R, 31, 41R, 51, 61, 71R, 81, 91

Volatility: 6, 16R, 26, 36R, 46, 56R, 66, 76R, 86, 96

Agreeableness

Compassion: 2R,12, 22, 32R, 42, 52R, 62R, 72, 82R, 92

Politeness: 7, 17R, 27, 37R, 47, 57, 67R, 77R, 87R, 97R

Conscientiousness

Industriousness: 3, 13R, 23R, 33R, 43, 53R, 63, 73, 83R, 93R

Orderliness: 8R, 18, 28, 38, 48R, 58, 68R, 78R, 88, 98

Extraversion

Enthusiasm: 4, 14R, 24R, 34R, 44, 54R, 64R, 74, 84, 94

Assertiveness: 9, 19, 29R, 39, 49R, 59, 69, 79R, 89, 99R

Openness/Intellect

Intellect: 5, 15R, 25, 35, 45R, 55R, 65, 75, 85R, 95

Openness: 10, 20, 30, 40, 50R, 60R, 70, 80R, 90R, 100

Reverse response scores for items followed by “R” (i.e. 1=5, 2=4, 4=2, 5=1). To compute scale scores, average completed items within each scale. To compute Big Five scores, average scores for the two aspects within each domain.

Reference: DeYoung, C. G., Quilty, L. C., & Peterson, J. B. (2007). Between facets and domains: 10 Aspects of the Big Five. Journal of Personality and Social Psychology, 93, 880-896.

# Discrete Emotion Questionnaire

## Reaction DEQ

While watching the previous video, to what degree did you experience any of the following emotions?

Anger

Disgust

Fear

Joy

Sadness

Surprise

Be as honest as possible, but rely on your initial feeling and do not think too much about each item.

*Use the following scale:*

1 = Not at all

2 = A Small Amount

3 = A Moderate Amount

4 = A High Amount

5 = An Extreme Amount

## Baseline DEQ

To what degree did you experience any of the following emotions today?

Anger

Disgust

Fear

Joy

Sadness

Surprise

Be as honest as possible, but rely on your initial feeling and do not think too much about each item.

*Use the following scale:*

1 = Not at all

2 = A Small Amount

3 = A Moderate Amount

4 = A High Amount

5 = An Extreme Amount

# Audio Task

## Audio Description

In this stage, you are asked to talk for a period of 60 seconds about how you felt whilst watching the previous video. Feel free to talk about anything related to the video (e.g. whether you liked it or not, any emotional experiences, feelings on the characters).

Please ensure the following before you begin this task that a) your microphone is on and functional and b) there is no background noise (e.g. television, music, other people) that can be picked up by your microphone.

## Audio Prompt

Please talk about your experience watching the previous video via your microphone for 60 seconds. Once you are ready to begin, click "Begin Recording".

Potential talking points:

Did you experience any particular emotions when watching the video?

If so, what emotions?

What aspects of the video caused you to feel those emotions?

Did you enjoy or not enjoy the video?

Did you find the video engaging?

Have you seen the video clip before?

If so, did you experience a different reaction to it this time?

If you experience any problems with the recording, please indicate so in the text box.

# Cognitive Reflection Test

Throughout the study, you will be asked to answer several cognitive questions. Do your best to reach the correct answer.

- ***CRT1***. If a bat and a ball cost $1.10 and the bat costs $1 more than the ball, how much does the ball cost? [Intuitive answer = 10 cent; Correct answer = 5 cent]
- ***CRT2***. If it takes 5 minutes for 5 machines to make 5 widgets, then how long would it take 100 machines to make 100 widgets? [Intuitive answer = 100 minutes; correct answer = 5 minutes]
- ***CRT3***. In a lake, there is a patch of lily pads. Every day, the patch doubles in size. If it takes 48 days for the patch to cover the entire lake, how long would it take for the patch to cover half of the lake? [Intuitive answer = 24 days; correct answer = 47 days]
- ***CRT4***. If John can drink one barrel of water in 6 days, and Mary can drink one barrel of water in 12 days, how long would it take them to drink one barrel of water together? [Intuitive answer = 9 days; correct answer = 4 days]
- ***CRT5***. A man buys a pig for $60, sells it for $70, buys it back for $80, and sells it finally for $90. How much has he made? [Intuitive answer = $10; correct answer = $20]
- ***CRT6***. Jerry received both the 15th highest and the 15th lowest mark in the class. How many students are in the class? [intuitive answer = 30 students; correct answer = 29 students]
- ***CRT7***. Simon decided to invest $8,000 in the stock market one day early in 2008. Six months after he invested, on July 17, the stocks he had purchased were down 50%. Fortunately for Simon, from July 17 to October 17, the stocks he had purchased went up 75%. At this point, Simon has: a. broken even in the stock market, b. is ahead of where he began, c. has lost money [Intuitive answer = B; correct answer = C].

# Video Description (Table S3)

**Table S3**

*Description of Video Clips used in this Study.*

| **Emotion** | **Source** | **Video Clip Description** |
| --- | --- | --- |
| Anger | Schindler’s List | A Nazi guard shoots concentration camp prisoners with a rifle. |
| Disgust | Trainspotting | A man must retrieve pills from a dirty toilet. |
| Fear | Annabelle | A woman is trapped in a basement with a monster. |
| Joy | When Harry Met Sally | A man and woman talk about gender differences |
|  |  | in sexual relationships and intimacy in a public diner. |
| Sadness | The Lion King | A cub is saved at the cost of his father’s life. |
|  |  | The cub grieves the death of his father. |
| Surprise | Test Your Awareness: Who Dunnit? | A murder is investigated. Once the investigator has |
|  |  | identified the murderer, he asks the viewer did they spot |
|  |  | the 21 differences made in the background during the scene. |
