# Supplementary material for: Investigating the Relationships Between Basic Emotions and the Big Five Personality Traits and Their Sub‐Traits
Source: J Pers. 2025 May 15;94(2):237–51. doi: 10.1111/jopy.13027 (PMC12988340; doi:10.1111/jopy.13027)
Supplement: Supplementary file 4 — Table S4. The results of regression models where each Big Five trait was entered as simultaneous predictors for each baseline emotion. [file JOPY-94-237-s002.docx]

**Table S4**

*The results of regression models where each Big Five trait was entered as simultaneous predictors for each baseline emotion.*

| **Outcome** | **Predictor(s)** | **b** | **SE** | **t** | **B** | **p** |
| --- | --- | --- | --- | --- | --- | --- |
| Anger  Baseline | (Intercept) | 0.972 | 0.656 | 1.482 | 0.000 | 0.140 |
|  | Openness to experience | -0.014 | 0.103 | -0.139 | -0.010 | 0.890 |
|  | Conscientiousness | -0.120 | 0.093 | -1.301 | -0.095 | 0.195 |
|  | **Extraversion** | **0.206** | **0.093** | **2.214** | **0.162** | **0.028*** |
|  | Agreeableness | -0.137 | 0.101 | -1.351 | -0.096 | 0.178 |
|  | **Neuroticism** | **0.311** | **0.077** | **4.034** | **0.303** | **0.001***** |
| **Outcome** | **Predictor(s)** | **b** | **SE** | **t** | **B** | **p** |
| Disgust  Baseline | (Intercept) | 2.396 | 0.610 | 3.929 | 0.000 | 0.001*** |
|  | Openness to experience | -0.024 | 0.095 | -0.255 | -0.019 | 0.799 |
|  | Conscientiousness | -0.037 | 0.086 | -0.433 | -0.033 | 0.666 |
|  | Extraversion | 0.063 | 0.087 | 0.728 | 0.055 | 0.467 |
|  | **Agreeableness** | **-0.318** | **0.094** | **-3.371** | **-0.245** | **0.001***** |
|  | Neuroticism | 0.069 | 0.072 | 0.960 | 0.074 | 0.338 |
|  |  |  |  |  |  |  |
| **Outcome** | **Predictor(s)** | **b** | **SE** | **t** | **B** | **p** |
| Fear  Baseline | (Intercept) | 0.234 | 0.721 | 0.324 | - 0.000 | 0.746 |
|  | Openness to experience | 0.019 | 0.113 | 0.165 | 0.012 | 0.869 |
|  | Conscientiousness | -0.028 | 0.102 | -0.271 | 0.020 | 0.786 |
|  | Extraversion | -0.132 | 0.102 | -1.290 | -0.094 | 0.199 |
|  | **Agreeableness** | **0.225** | **0.111** | **2.016** | **0.142** | **0.045*** |
|  | **Neuroticism** | **0.304** | **0.085** | **3.594** | **0.269** | **0.001***** |
|  |  |  |  |  |  |  |
| **Outcome** | **Predictor(s)** | **b** | **SE** | **t** | **Β** | **p** |
| Joy  Baseline | (Intercept) | 1.839 | 0.853 | 2.155 | 0.000 | 0.032* |
|  | Openness to experience | -0.159 | 0.133 | -1.195 | -0.088 | 0.234 |
|  | Conscientiousness | 0.028 | 0.120 | 0.232 | 0.017 | 0.817 |
|  | **Extraversion** | **0.423** | **0.121** | **3.492** | **0.260** | **0.001***** |
|  | Agreeableness | 0.158 | 0.132 | 1.201 | 0.086 | 0.231 |
|  | Neuroticism | -0.079 | 0.100 | -0.785 | -0.060 | 0.433 |
|  |  |  |  |  |  |  |

**Tables S4** (continued).

| **Outcome** | **Predictor(s)** | **b** | **SE** | **t** | **Β** | **p** |
| --- | --- | --- | --- | --- | --- | --- |
| Sadness  Baseline | (Intercept) | 1.349 | 0.814 | 1.656 | 0.000 | 0.099 |
|  | Openness to Experience | 0.137 | 0.127 | 1.079 | 0.076 | 0.282 |
|  | **Conscientiousness** | **-0.455** | **0.115** | **-3.964** | **-0.280** | **0.001***** |
|  | Extraversion | 0.051 | 0.116 | 0.440 | 0.031 | 0.660 |
|  | Agreeableness | 0.123 | 0.126 | 0.979 | 0.067 | 0.329 |
|  | **Neuroticism** | **0.297** | **0.096** | **3.107** | **0.225** | **0.002*** |

| **Outcome** | **Predictor(s)** | **b** | **SE** | **t** | **B** | **p** |
| --- | --- | --- | --- | --- | --- | --- |
| Surprise Baseline | (Intercept) | 1.502 | 0.946 | 1.587 | 0.000 | 0.114 |
|  | Openness to Experience | 0.126 | 0.148 | 0.852 | 0.065 | 0.395 |
|  | Conscientiousness | -0.071 | 0.133 | -0.530 | -0.041 | 0.597 |
|  | Extraversion | 0.069 | 0.134 | 0.513 | 0.040 | 0.609 |
|  | Agreeableness | 0.073 | 0.146 | 0.498 | 0.037 | 0.619 |
|  | Neuroticism | -0.145 | 0.111 | -1.309 | -0.103 | 0.192 |
