# Supplementary material for: Investigating the Relationships Between Basic Emotions and the Big Five Personality Traits and Their Sub‐Traits
Source: J Pers. 2025 May 15;94(2):237–51. doi: 10.1111/jopy.13027 (PMC12988340; doi:10.1111/jopy.13027)
Supplement: Supplementary file 5 — Table S5. The results of regression models where each Big Five trait was entered as simultaneous predictors for each reaction emotion. [file JOPY-94-237-s007.docx]

**Table S5**

*The results of regression models where each Big Five trait was entered as simultaneous predictors for each reaction emotion.*

| **Outcome** | **Predictor(s)** | **b** | **SE** | **t** | **B** | **p** |
| --- | --- | --- | --- | --- | --- | --- |
| Anger  Reaction | (Intercept) | 0.348 | 0.450 | 0.773 | 0.000 | 0.441 |
|  | **Anger Baseline** | **0.198** | **0.049** | **4.071** | **0.283** | **0.001***** |
|  | Openness to Experience | -0.128 | 0.070 | -1.830 | -0.130 | 0.069 |
|  | **Conscientiousness** | **0.141** | **0.063** | **2.228** | **0.160** | **0.027*** |
|  | **Extraversion** | **0.157** | **0.064** | **2.444** | **0.177** | **0.015*** |
|  | **Agreeableness** | **0.138** | **0.070** | **1.980** | **0.137** | **0.049*** |
|  | Neuroticism | 0.082 | 0.055 | 1.503 | 0.115 | 0.135 |
| **Outcome** | **Predictor(s)** | **b** | **SE** | **t** | **B** | **p** |
| Disgust  Reaction | (Intercept) | 0.923 | 0.539 | 1.713 | 0.000 | 0.088 |
|  | Disgust Baseline | 0.018 | 0.061 | 0.296 | 0.021 | 0.768 |
|  | Openness to Experience | -0.099 | 0.081 | 1.218 | -0.091 | 0.225 |
|  | Conscientiousness | 0.050 | 0.073 | 0.689 | 0.052 | 0.492 |
|  | **Extraversion** | **0.175** | **0.074** | **2.373** | **0.178** | **0.019*** |
|  | **Agreeableness** | **0.195** | **0.082** | **2.371** | **0.176** | **0.019*** |
|  | **Neuroticism** | **0.149** | **0.061** | **2.442** | **0.188** | **0.015*** |
|  |  |  |  |  |  |  |
| **Outcome** | **Predictor(s)** | **b** | **SE** | **t** | **B** | **p** |
| Fear  Reaction | (Intercept) | 0.022 | 0.489 | 0.045 | 0.000 | 0.964 |
|  | **Fear Baseline** | **0.126** | **0.048** | **2.611** | **0.178** | **0.010*** |
|  | Openness to Experience | -0.124 | 0.076 | 1.625 | -0.113 | 0.106 |
|  | Conscientiousness | 0.012 | 0.069 | 0.177 | 0.012 | 0.860 |
|  | **Extraversion** | **0.201** | **0.070** | **2.886** | **0.203** | **0.004*** |
|  | **Agreeableness** | **0.282** | **0.076** | **3.689** | **0.252** | **0.001***** |
|  | **Neuroticism** | **0.196** | **0.059** | **3.315** | **0.245** | **0.001***** |
|  |  |  |  |  |  |  |
| **Outcome** | **Predictor(s)** | **b** | **SE** | **t** | **Β** | **p** |
| Joy  Reaction | (Intercept) | 2.197 | 0.497 | 4.420 | 0.000 | 0.001*** |
|  | **Joy Baseline** | **0.086** | **0.041** | **2.095** | **0.145** | **0.037*** |
|  | Openness to Experience | 0.000 | 0.077 | 0.000 | 0.000 | 1.000 |
|  | **Conscientiousness** | **-0.262** | **0.069** | **3.781** | **-0.274** | **0.001***** |
|  | **Extraversion** | **0.212** | **0.072** | **2.945** | **0.220** | **0.004*** |
|  | Agreeableness | -0.106 | 0.076 | 1.387 | -0.098 | 0.167 |
|  | Neuroticism | 0.053 | 0.058 | 0.924 | 0.069 | 0.357 |
|  |  |  |  |  |  |  |

**Tables S5** (continued).

| **Outcome** | **Predictor(s)** | **b** | **SE** | **t** | **B** | **p** |
| --- | --- | --- | --- | --- | --- | --- |
| Sadness  Reaction | (Intercept) | 0.175 | 0.467 | 0.374 | 0.000 | 0.709 |
|  | **Sadness Baseline** | **0.153** | **0.041** | **3.770** | **0.268** | **0.001***** |
|  | Openness to Experience | -0.071 | 0.073 | 0.982 | -0.069 | 0.327 |
|  | Conscientiousness | 0.107 | 0.068 | 1.577 | 0.116 | 0.116 |
|  | **Extraversion** | **0.141** | **0.066** | **2.149** | **0.152** | **0.033*** |
|  | **Agreeableness** | **0.222** | **0.072** | **3.092** | **0.212** | **0.002*** |
|  | **Neuroticism** | **0.191** | **0.074** | **2.577** | **0.191** | **0.011*** |
|  |  |  |  |  |  |  |
| **Outcome** | **Predictor(s)** | **b** | **SE** | **t** | **Β** | **p** |
| Surprise Reaction | (Intercept) | 1.357 | 0.745 | 1.821 | 0.000 | 0.070* |
|  | **Surprise Baseline** | **0.115** | **0.056** | **2.062** | 0.142 | **0.040*** |
|  | **Openness to Experience** | **-0.243** | **0.116** | **2.098** | -0.156 | **0.037*** |
|  | Conscientiousness | 0.009 | 0.104 | 0.090 | 0.007 | 0.929 |
|  | **Extraversion** | **0.191** | **0.105** | **1.821** | 0.136 | **0.070*** |
|  | **Agreeableness** | **0.264** | **0.114** | **2.302** | 0.166 | **0.022*** |
|  | **Neuroticism** | **0.183** | **0.087** | **2.092** | 0.161 | **0.038*** |
