# Supplementary material for: Investigating the Relationships Between Basic Emotions and the Big Five Personality Traits and Their Sub‐Traits
Source: J Pers. 2025 May 15;94(2):237–51. doi: 10.1111/jopy.13027 (PMC12988340; doi:10.1111/jopy.13027)
Supplement: Supplementary file 6 — Table S6. The results of regression models where each sub‐trait was entered as simultaneous predictors for each baseline emotion. [file JOPY-94-237-s006.docx]

**Table S6**

*The results of regression models where each sub-trait was entered as simultaneous predictors for each baseline emotion*

| **Outcome** | | **Predictor(s)** | **b** | **SE** | **t** | **B** | **p** |
| --- | --- | --- | --- | --- | --- | --- | --- |
| Anger  Baseline | | (Intercept) | 1.243 | 0.753 | 1.651 | 0.000 | 0.100 |
|  |  | Openness | -0.039 | 0.093 | -0.417 | -0.034 | 0.677 |
|  |  | Intellect | -0.039 | 0.102 | -0.386 | -0.034 | 0.700 |
|  |  | Industriousness | -0.149 | 0.119 | -1.247 | -0.136 | 0.214 |
|  |  | Orderliness | 0.002 | 0.093 | 0.022 | 0.002 | 0.983 |
|  |  | **Assertiveness** | **0.215** | **0.101** | **2.135** | **0.211** | **0.034*** |
|  |  | Enthusiasm | -0.018 | 0.108 | -0.171 | -0.016 | 0.864 |
|  |  | Compassion | 0.006 | 0.112 | 0.053 | 0.005 | 0.958 |
|  |  | Politeness | -0.099 | 0.123 | -0.805 | -0.075 | 0.422 |
|  |  | Withdrawal | 0.181 | 0.123 | 1.467 | 0.182 | 0.144 |
|  |  | Volatility | 0.104 | 0.093 | 1.117 | 0.116 | 0.266 |
|  | |  |  |  |  |  |  |
| **Outcome** | **Predictor(s)** | **b** | **SE** | **t** | **B** | **p** |  |
| Disgust  Baseline | (Intercept) | 2.827 | 0.699 | 4.043 | 0.000 | 0.001* |  |
|  | Openness | 0.023 | 0.087 | 0.265 | 0.022 | 0.791 |  |
|  | Intellect | -0.027 | 0.094 | -0.289 | -0.026 | 0.773 |  |
|  | Industriousness | -0.067 | 0.111 | -0.601 | -0.067 | 0.548 |  |
|  | Orderliness | 0.060 | 0.086 | 0.689 | 0.059 | 0.492 |  |
|  | Assertiveness | -0.095 | 0.094 | -1.018 | -0.103 | 0.310 |  |
|  | Enthusiasm | 0.101 | 0.100 | 1.016 | 0.097 | 0.311 |  |
|  | Compassion | -0.111 | 0.104 | -1.064 | -0.112 | 0.289 |  |
|  | **Politeness** | **-0.264** | **0.114** | **-2.312** | **-0.220** | **0.022*** |  |
|  | Withdrawal | -0.065 | 0.114 | -0.569 | -0.072 | 0.570 |  |
|  | Volatility | 0.050 | 0.086 | 0.585 | 0.062 | 0.559 |  |

| **Outcome** | **Predictor(s)** | **b** | **SE** | **t** | **B** | **p** |
| --- | --- | --- | --- | --- | --- | --- |
| Fear  Baseline | (Intercept) | -0.239 | 0.804 | -0.297 | 0.000 | 0.767 |
|  | Openness | -0.072 | 0.100 | -0.721 | -0.057 | 0.472 |
|  | Intellect | 0.135 | 0.109 | 1.246 | 0.106 | 0.214 |
|  | Industriousness | -0.188 | 0.127 | -1.480 | -0.156 | 0.141 |
|  | Orderliness | 0.103 | 0.099 | 1.040 | 0.084 | 0.300 |
|  | Assertiveness | 0.054 | 0.108 | 0.500 | 0.048 | 0.618 |
|  | Enthusiasm | 0.007 | 0.115 | 0.064 | 0.006 | 0.949 |
|  | Compassion | -0.016 | 0.120 | -0.133 | -0.013 | 0.895 |
|  | Politeness | 0.135 | 0.131 | 1.033 | 0.093 | 0.303 |
|  | **Withdrawal** | **0.499** | **0.132** | **3.798** | **0.456** | **0.001***** |
|  | Volatility | -0.122 | 0.099 | -1.229 | -0.124 | 0.221 |

**Table S6** (continued).

| **Outcome** | **Predictor(s)** | **b** | **SE** | **t** | **B** | **p** |
| --- | --- | --- | --- | --- | --- | --- |
| Joy  Baseline | (Intercept) | 1.643 | 0.974 | 1.686 | 0.000 | 0.093 |
|  | Openness | -0.029 | 0.121 | -0.240 | -0.020 | 0.811 |
|  | Intellect | 0.004 | 0.132 | 0.027 | 0.002 | 0.978 |
|  | Industriousness | -0.003 | 0.154 | -0.022 | -0.002 | 0.982 |
|  | Orderliness | 0.076 | 0.120 | 0.633 | 0.053 | 0.528 |
|  | Assertiveness | -0.004 | 0.130 | -0.032 | -0.003 | 0.975 |
|  | **Enthusiasm** | **0.495** | **0.139** | **3.560** | **0.335** | **0.001*** |
|  | Compassion | -0.048 | 0.145 | -0.333 | -0.034 | 0.740 |
|  | Politeness | 0.015 | 0.159 | 0.093 | 0.009 | 0.926 |
|  | Withdrawal | 0.033 | 0.159 | 0.208 | 0.026 | 0.835 |
|  | Volatility | -0.120 | 0.120 | -0.995 | -0.105 | 0.321 |

| **Outcome** | **Predictor(s)** | **b** | **SE** | **t** | **Β** | **p** |  |
| --- | --- | --- | --- | --- | --- | --- | --- |
| Sadness  Baseline | | (Intercept) | 1.265 | 0.935 | 1.353 | 0.000 | 0.178 |
|  |  | Openness | 0.143 | 0.116 | 1.237 | 0.098 | 0.218 |
|  |  | Intellect | -0.027 | 0.126 | -0.212 | -0.018 | 0.832 |
|  |  | Industriousness | -0.134 | 0.148 | -0.908 | -0.096 | 0.365 |
|  |  | **Orderliness** | **-0.249** | **0.116** | **-2.154** | **-0.174** | **0.032*** |
|  |  | Assertiveness | 0.092 | 0.125 | 0.735 | 0.070 | 0.463 |
|  |  | Enthusiasm | 0.003 | 0.134 | 0.021 | 0.002 | 0.983 |
|  |  | Compassion | 0.078 | 0.139 | 0.558 | 0.055 | 0.577 |
|  |  | Politeness | -0.052 | 0.152 | -0.341 | -0.031 | 0.734 |
|  |  | **Withdrawal** | **0.360** | **0.153** | **2.354** | **0.282** | **0.020*** |
|  |  | Volatility | -0.015 | 0.115 | -0.131 | -0.013 | 0.896 |
|  | |  |  |  |  |  |  |
| **Outcome** | | **Predictor(s)** | **b** | **SE** | **t** | **B** | **p** |
| Surprise  Baseline | | (Intercept) | 1.918 | 1.086 | 1.767 | 0.000 | 0.079 |
|  |  | Openness | 0.030 | 0.135 | 0.224 | 0.019 | 0.823 |
|  |  | Intellect | 0.141 | 0.147 | 0.964 | 0.089 | 0.336 |
|  |  | Industriousness | -0.070 | 0.172 | -0.406 | -0.047 | 0.685 |
|  |  | Orderliness | 0.047 | 0.134 | 0.352 | 0.031 | 0.725 |
|  |  | Assertiveness | -0.142 | 0.145 | -0.980 | -0.102 | 0.328 |
|  |  | Enthusiasm | 0.162 | 0.155 | 1.045 | 0.102 | 0.297 |
|  |  | Compassion | 0.117 | 0.162 | 0.723 | 0.078 | 0.470 |
|  |  | Politeness | -0.184 | 0.177 | -1.037 | -0.101 | 0.301 |
|  |  | Withdrawal | -0.007 | 0.178 | -0.037 | -0.005 | 0.971 |
|  |  | Volatility | -0.188 | 0.134 | -1.403 | -0.154 | 0.162 |
|  |  |  |  |  |  |  |  |
