# Supplementary material for: Investigating the Relationships Between Basic Emotions and the Big Five Personality Traits and Their Sub‐Traits
Source: J Pers. 2025 May 15;94(2):237–51. doi: 10.1111/jopy.13027 (PMC12988340; doi:10.1111/jopy.13027)
Supplement: Supplementary file 7 — Table S7. The results of regression models where each sub‐trait was entered as simultaneous predictors for each reaction emotion. [file JOPY-94-237-s003.docx]

**Table S7**

*The results of regression models where each sub-trait was entered as simultaneous predictors for each reaction emotion*

| **Outcome** | | **Predictor(s)** | **b** | **SE** | **t** | **B** | **p** |
| --- | --- | --- | --- | --- | --- | --- | --- |
| Anger  Reaction | | (Intercept) | 0.486 | 0.521 | 0.933 | 0.000 | 0.352 |
|  |  | **Anger Baseline** | **0.196** | **0.050** | **3.954** | **0.281** | **0.001*** |
|  |  | Openness | -0.040 | 0.064 | -0.619 | -0.050 | 0.537 |
|  |  | Intellect | -0.099 | 0.070 | -1.414 | -0.123 | 0.159 |
|  |  | Industriousness | 0.084 | 0.082 | 1.019 | 0.110 | 0.309 |
|  |  | Orderliness | 0.082 | 0.064 | 1.279 | 0.105 | 0.202 |
|  |  | Assertiveness | 0.071 | 0.070 | 1.015 | 0.100 | 0.311 |
|  |  | Enthusiasm | 0.071 | 0.074 | 0.955 | 0.087 | 0.341 |
|  |  | Compassion | 0.101 | 0.077 | 1.316 | 0.132 | 0.190 |
|  |  | Politeness | 0.011 | 0.085 | 0.130 | 0.012 | 0.897 |
|  |  | Withdrawal | 0.054 | 0.085 | 0.638 | 0.078 | 0.524 |
|  |  | Volatility | 0.015 | 0.064 | 0.233 | 0.024 | 0.816 |
|  | |  |  |  |  |  |  |
| **Outcome** | **Predictor(s)** | **b** | **SE** | **t** | **B** | **p** |  |
| Disgust  Reaction | (Intercept) | 1.073 | 0.625 | 1.718 | 0.000 | 0.087 |  |
|  | Disgust Baseline | 0.018 | 0.062 | 0.294 | 0.021 | 0.769 |  |
|  | Openness | -0.040 | 0.074 | -0.536 | -0.045 | 0.593 |  |
|  | Intellect | -0.087 | 0.081 | -1.072 | -0.097 | 0.285 |  |
|  | Industriousness | 0.027 | 0.095 | 0.287 | 0.032 | 0.775 |  |
|  | Orderliness | 0.035 | 0.074 | 0.472 | 0.041 | 0.638 |  |
|  | Assertiveness | 0.116 | 0.080 | 1.437 | 0.147 | 0.152 |  |
|  | Enthusiasm | 0.041 | 0.086 | 0.472 | 0.045 | 0.637 |  |
|  | Compassion | 0.143 | 0.090 | 1.600 | 0.169 | 0.111 |  |
|  | Politeness | 0.055 | 0.099 | 0.555 | 0.054 | 0.580 |  |
|  | Withdrawal | 0.085 | 0.098 | 0.861 | 0.110 | 0.390 |  |
|  | Openness | -0.040 | 0.074 | -0.536 | 0.077 | 0.593 |  |

| **Outcome** | **Predictor(s)** | **b** | **SE** | **t** | **B** | **p** |
| --- | --- | --- | --- | --- | --- | --- |
| Fear  Reaction | (Intercept) | 0.270 | 0.559 | 0.483 | 0.000 | 0.629 |
|  | **Fear Baseline** | **0.134** | **0.050** | **2.669** | **0.189** | **0.008*** |
|  | Openness | -0.015 | 0.069 | -0.214 | -0.017 | 0.831 |
|  | Intellect | -0.132 | 0.076 | -1.744 | -0.147 | 0.083 |
|  | Industriousness | 0.131 | 0.089 | 1.472 | 0.153 | 0.143 |
|  | Orderliness | -0.037 | 0.069 | -0.536 | -0.043 | 0.592 |
|  | Assertiveness | 0.054 | 0.075 | 0.723 | 0.068 | 0.471 |
|  | Enthusiasm | 0.083 | 0.080 | 1.040 | 0.092 | 0.299 |
|  | **Compassion** | **0.241** | **0.083** | **2.888** | **0.280** | **0.004*** |
|  | Politeness | -0.023 | 0.091 | -0.248 | -0.022 | 0.804 |
|  | Withdrawal | 0.149 | 0.095 | 1.573 | 0.192 | 0.117 |
|  | Volatility | 0.044 | 0.069 | 0.635 | 0.063 | 0.526 |

**Table S7** (continued).

| **Outcome** | **Predictor(s)** | **b** | **SE** | **t** | **B** | **p** |
| --- | --- | --- | --- | --- | --- | --- |
| Joy  Reaction | (Intercept) | 1.929 | 0.567 | 3.402 | 0.000 | 0.001 |
|  | **Joy Baseline** | **0.093** | **0.042** | **2.224** | **0.157** | **0.027*** |
|  | Openness | -0.043 | 0.070 | -0.618 | -0.050 | 0.537 |
|  | Intellect | 0.022 | 0.076 | 0.293 | 0.026 | 0.769 |
|  | Industriousness | -0.053 | 0.089 | -0.590 | -0.064 | 0.556 |
|  | **Orderliness** | **-0.211** | **0.070** | **-3.037** | **-0.251** | **0.003*** |
|  | **Assertiveness** | **0.182** | **0.075** | **2.420** | **0.237** | **0.016*** |
|  | Enthusiasm | 0.044 | 0.083 | 0.530 | 0.050 | 0.597 |
|  | Compassion | -0.043 | 0.084 | -0.514 | -0.052 | 0.608 |
|  | Politeness | -0.041 | 0.092 | -0.445 | -0.041 | 0.657 |
|  | Withdrawal | 0.148 | 0.092 | 1.609 | 0.197 | 0.109 |
|  | Volatility | -0.020 | 0.070 | -0.290 | -0.030 | 0.772 |

| **Outcome** | **Predictor(s)** | **b** | **SE** | **t** | **Β** | **p** |
| --- | --- | --- | --- | --- | --- | --- |
| Sadness  Reaction | (Intercept) | 0.251 | 0.533 | **0.471** | 0.000 | 0.638 |
|  | **Sadness Baseline** | **0.148** | **0.041** | **3.603** | **0.259** | **0.001***** |
|  | Openness | -0.020 | 0.066 | -0.305 | -0.024 | 0.761 |
|  | Intellect | -0.065 | 0.072 | -0.902 | -0.077 | 0.368 |
|  | **Industriousness** | **0.195** | **0.084** | **2.312** | **0.243** | **0.022*** |
|  | Orderliness | -0.026 | 0.066 | -0.395 | -0.032 | 0.693 |
|  | Assertiveness | 0.034 | 0.071 | 0.480 | 0.046 | 0.632 |
|  | Enthusiasm | 0.055 | 0.076 | 0.730 | 0.065 | 0.466 |
|  | Compassion | 0.187 | 0.079 | 2.368 | 0.233 | 0.019 |
|  | Politeness | -0.004 | 0.087 | -0.050 | -0.004 | 0.960 |
|  | Withdrawal | 0.140 | 0.088 | 1.585 | 0.192 | 0.115 |
|  | Volatility | 0.035 | 0.066 | 0.534 | 0.054 | 0.594 |
|  |  |  |  |  |  |  |
| **Outcome** | **Predictor(s)** | **b** | **SE** | **t** | **B** | **p** |
| Surprise  Reaction | (Intercept) | 0.942 | **0.845** | **1.114** | 0.000 | 0.267 |
|  | **Surprise Baseline** | **0.123** | **0.056** | **2.203** | **0.152** | **0.029*** |
|  | Openness | 0.031 | 0.104 | 0.301 | 0.025 | 0.764 |
|  | **Intellect** | **-0.313** | **0.114** | **-2.753** | -**0.244** | **0.006*** |
|  | Industriousness | 0.209 | 0.133 | 1.577 | 0.173 | 0.116 |
|  | Orderliness | -0.102 | 0.104 | -0.987 | -0.083 | 0.325 |
|  | **Assertiveness** | **0.241** | **0.113** | **2.144** | **0.214** | **0.033*** |
|  | Enthusiasm | 0.029 | 0.120 | 0.244 | 0.023 | 0.808 |
|  | Compassion | 0.095 | 0.125 | 0.756 | 0.078 | 0.450 |
|  | Politeness | 0.092 | 0.137 | 0.671 | 0.063 | 0.503 |
|  | **Withdrawal** | **0.329** | **0.137** | **2.393** | **0.298** | **0.018*** |
|  | Volatility | -0.045 | 0.104 | -0.434 | -0.046 | 0.665 |
|  |  |  |  |  |  |  |
